# Self-supervised machine learning pushes the sensitivity limit in label-free detection of single proteins below 10 kDa

**DOI:** 10.1038/s41592-023-01778-2

**Published:** 2023-02-27

**Authors:** Mahyar Dahmardeh, Houman Mirzaalian Dastjerdi, Hisham Mazal, Harald Köstler, Vahid Sandoghdar

**Affiliations:** 1 Max Planck Institute for the Science of Light, Erlangen, Germany; 2 Max-Planck-Zentrum für Physik und Medizin, Erlangen, Germany; 3 Department of Computer Science, Friedrich-Alexander-Universität Erlangen-Nürnberg, Erlangen, Germany; 4 Erlangen National High Performance Computing Center (NHR@FAU), Erlangen, Germany; 5 Department of Physics, Friedrich-Alexander-Universität Erlangen-Nürnberg, Erlangen, Germany

**Keywords:** Nanoscale biophysics, Proteomics

## Abstract

Interferometric scattering (iSCAT) microscopy is a label-free optical method capable of detecting single proteins, localizing their binding positions with nanometer precision, and measuring their mass. In the ideal case, iSCAT is limited by shot noise such that collection of more photons should extend its detection sensitivity to biomolecules of arbitrarily low mass. However, a number of technical noise sources combined with speckle-like background fluctuations have restricted the detection limit in iSCAT. Here, we show that an unsupervised machine learning isolation forest algorithm for anomaly detection pushes the mass sensitivity limit by a factor of 4 to below 10 kDa. We implement this scheme both with a user-defined feature matrix and a self-supervised FastDVDNet and validate our results with correlative fluorescence images recorded in total internal reflection mode. Our work opens the door to optical investigations of small traces of biomolecules and disease markers such as α-synuclein, chemokines and cytokines.

## Abstract (web summary)

An unsupervised machine learning approach for anomaly detection, implemented as both a user-defined feature matrix and a self-supervised deep neural network, improves the mass sensitivity of iSCAT by a factor of 4 to below 10 kDa.

## Main

Analysis of nanometer-scale matter is of the utmost importance for a variety of biomedical investigations^1–5^. During the last 100 years many clever techniques have been invented for characterization of macromolecules, for example, to resolve structure, map dynamics, assess chemical composition, and measure physical quantities such as size and weight. Methods based on nuclear magnetic resonance spectroscopy, electrophoresis, mass spectrometry, electron microscopy, fluorescence imaging and plasmon resonance spectroscopy have introduced decisive information, but each approach also has its limitations. Thus, new innovations are continuously sought to push the measurement boundaries. Optical methods are desirable in this quest because they can be non-invasive and compatible with real-time studies. Indeed, the optical cross-section of matter is intrinsically large enough to enable the detection of single molecules and proteins in direct extinction measurements^6–8^, in which the incident field (or a fraction of it) interferes with the tiny amount of light that is coherently scattered by the nano-object of interest^9–11^.

The interferometric signal that is generated by the scattered light (iSCAT) not only enables the detection and sensing of sub-wavelength nanoparticles such as single proteins, but it also provides information on the particle size^8^. Indeed, iSCAT measurements have recently been calibrated to determine protein mass^11,12^, leading to a technology that is now also offered commercially (Refeyn Ltd., Oxford, UK). Given that the sensitivity of iSCAT is ultimately limited by shot noise^10,13,14^, one could expect to detect an arbitrarily small amount of matter if only one could collect a sufficiently large number of photons. In practice, however, the dynamics of residual background fluctuations prevents one from reaching this ideal situation^14^, and hence proteins lighter than approximately 40 kDa have not been detected^11^. In this work, we report on a substantial improvement in iSCAT detection sensitivity to the range of 9 kDa using machine learning approaches for anomaly detection^15^. We benchmark and validate our results by performing fluorescence detection in total internal reflection (TIRF) mode. More sophisticated analysis of the signal and background might enable the sensitivity limit to be extended even further in the near future.

## The iSCAT signal

**Fig. 1:**
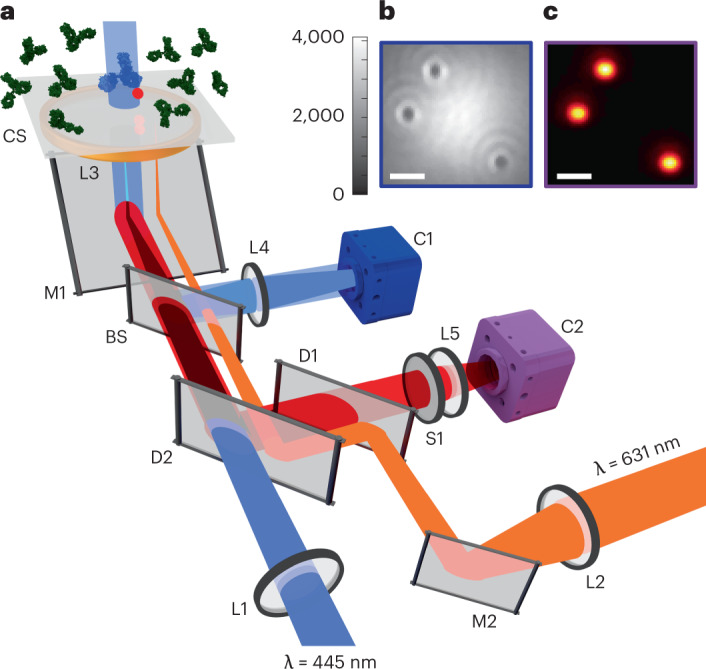
Optical set-up. **a**, Schematic diagram of the optical set-up. A laser beam at 445 nm is focused at the back focal plane of an oil-immersion objective to generate a quasi-collimated beam with a diameter of approximately 6 μm at the sample surface. The sample chamber consists of a microscope coverglass sealed to the bottom of a plexiglass dish containing 2 ml buffer solution. L1, L2, L3, L4 and L5 denote the lenses, including the microscope objective. CS, BS and M1 signify the coverglass, beam splitter and the objective coupling mirror, respectively. D1, D2, S1 and M2 show the dichroic filters and the mirror used for guiding the TIRF beam. C1 and C2 represent the iSCAT and TIRF imaging cameras, respectively. **b**, Single-frame, experimental iSCAT image of 100 nm fluorescent beads deposited on the coverglass. Color bar shows the camera bit readout. **c**, Single-frame TIRF image of the same beads as in **b**. Scale bars, 1.5 μm.

Figure 1a shows the iSCAT sensing set-up. A laser beam centered at a wavelength of 445 nm illuminates a sample that consists of an aqueous buffer on a microscope coverglass. A fraction of the incident light is reflected at this interface and is used as the reference in its interference with the scattered light from the nano-object under study^10,13^. The detected optical power on the camera is

1 $${P}_{{{{\rm{d}}}}}\propto | {E}_{{{{\rm{r}}}}}{| }^{2}+| {E}_{{{{\rm{s}}}}}{| }^{2}+2| {E}_{{{{\rm{r}}}}}| | {E}_{{{{\rm{s}}}}}| \cos \phi ,$$

where *E*_r_ = *rE*_i_, *E*_s_ = *sE*_i_ and *E*_i_ denote the electric fields of the reference, scattered and incident light fields, respectively, and *ϕ* signifies the phase difference between the latter two quantities. To add fluorescence imaging capabilities, a laser beam centered at a wavelength of 631 nm is used to illuminate the sample in TIRF mode through the same microscope objective. The fluorescence signal is filtered through a dichroic mirror and is imaged on a second camera. Figure 1b shows an example of the iSCAT image for 100 nm polymer beads bound to the coverglass. In Fig. 1c we show the TIRF image of the same beads, which contained fluorescent dyes.

The scattered field of a nanoparticle is proportional to the incident field via its polarizability (*α*) so that *s* ∝ *α*. For small nano-objects, the intensity of the scattered light (∣*E*_s_∣^2^) becomes negligible compared with the other terms in equation (1). Hence, the iSCAT contrast (*C*) of a small particle can be formulated as

2 $$C=\frac{{P}_{{{{\rm{d}}}}}-{P}_{{{{\rm{r}}}}}}{{P}_{{{{\rm{r}}}}}} \sim 2\frac{| {E}_{{{{\rm{s}}}}}| }{| {E}_{{{{\rm{r}}}}}| }\cos \phi =2\frac{s}{r}\cos \phi \,,$$

where *P*_d_ and *P*_r_ refer to the detected and reference powers, respectively. Considering that *α* is proportional to the particle volume and assuming a constant density for protein matter, one can conclude that *C* is linearly proportional to the particle mass^8^. Thus, the iSCAT signal provides a measure for mass photometry^11,12^.

The iSCAT image in Fig. 1b was recorded in one frame in a short exposure time of 20 μs. Visualizing single proteins, however, requires longer integration times and an elaborate analysis to account for the speckle-like background features that are caused by coherent scattering from slight imperfections of the sample surface^14^. In brief, this analysis exploits the temporal change of the signal as a protein lands on the sensor substrate to eliminate the static background of the sample by comparing each video frame with its neighbors. In practice, a series of careful steps establish an algorithm that performs a differential rolling average (DRA) of several hundred camera frames, followed by the application of various tools to identify the point spread function (PSF) of individual proteins and determine their iSCAT contrasts^8,12^. It was found that the integration time for each protein event cannot be extended beyond a few seconds due to residual background dynamics. As a result, the detection sensitivity reaches a plateau at a molecular weight of approximately 40–50 kDa. The analysis procedure and its limitations are given in a recent publication^14^ as well as in Supplementary Information, Section 3.

## Machine learning

Computer vision and machine learning methods have recently been used in microscopy applications with an emphasis on correcting the background or enhancing the signal. For background correction, conventional computer vision methods have been used, exploiting temporal and spatial information in two independent steps^16,17^. In addition, scientists have applied supervised^18^ and unsupervised^19^ deep neural networks (DNNs) in machine learning. For example, supervised DNNs were used to extract spatiotemporal features in localization microscopy and particle tracking^20,21^. Supervised algorithms are, however, limited in scope because they require knowledge of the ground truth, which in turn implies full knowledge of the signal and noise properties. An example for getting around this restriction has recently been reported, in which an unsupervised DNN based on FastDVDnet^22^ was used to denoise an image series^23^.

In this work, we exploit self-supervised FastDVDnet in a different tailor-made scheme whereby we first denoise our images and then subtract the de-noised frame from the frame of interest to identify the PSFs of the rare landing proteins. Here, a frame *t* in the DRA video is analyzed by comparing it with its neighboring frames *t* − *k* and *t* + *k* with suitable stride *k* (Supplementary Table 1). Next, we classify the outcome using isolation forest (iForest)^24^, which is an unsupervised algorithm in anomaly detection. Anomaly detection encompasses a general class of algorithms in which one first establishes a ‘normal’ signal and then identifies deviations or ‘anomalies’. The normal signal in our experiment is the residual background speckle image obtained by averaging over multiple frames immediately before and after the frames that contain a protein landing event. The output of iForest thus becomes a vector of true (anomalous) and false (normal) values for each pixel. iForest has been successfully applied to computer vision, signal processing and communication applications^15,25–27^. We present a brief overview of various concepts relevant to our work in the Supplementary Information.

To gain more direct insight into the underlying physical criteria in anomaly detection, we also explore a user-defined approach in which one chooses a set of temporal (mean, standard deviation and so on) and spatial features (for example, PSF-like figures) that are evaluated for a certain pixel range in each image frame (Supplementary Figs. 3–8). A given frame *t* is then re-shaped for each feature into a one-dimensional vector with elements representing the pixel values of that frame. Next, a feature matrix is composed of the one-dimensional vectors that are produced from the aforementioned frames (Supplementary Figs. 8a, and 9a), and the resulting feature matrix is fed to iForest for classification. We note that the initial choice of the user-defined features is based on physical considerations such as the PSF size, typical DRA window and camera frame rate, which mostly depend on the optical system and not on the protein under study. In other words, the user can explore various options, which can be validated on independent data, for example, on larger proteins. Furthermore, the usefulness of the chosen features can be assessed in simulations of synthetic data. Nevertheless, the success and efficiency of the user-defined feature matrix depend on the aptitude and judgment of the user. Hence, we rely on the DNN for our final conclusions given that it does not require critical input from the user.

## Results

**Fig. 2:**
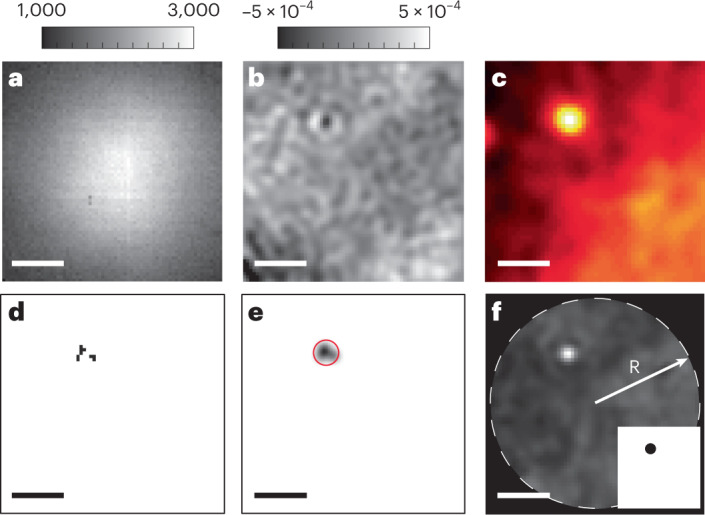
Benchmarking methods for BSA (66 kDa). **a**, An example of a single frame of raw iSCAT video for the landing event of a BSA molecule. The color bar shows the camera bit readout. **b**, The outcome of a standard DRA treatment of 1,500 neighboring frames. The PSF of one BSA protein is evident on a residual speckle-like background with the color bar depicting the iSCAT contrast, *C*. **c**, One frame of the raw TIRF video for the same protein landing event as in **a** and **b**. **d**, The corresponding hot pixels result from a user-defined anomaly detection analysis of the DRA outcome in **b**. **e**, Convolution of the hot pixels in **d** with the experimental PSF. The red circle marks the binary mask applied to the center mass of the distribution. **f**, The probability map of anomaly detection based on the DNN approach for the same DRA frame in **b**. R is the radius of the central circular part of the image that was considered in the analysis. The inset shows the corresponding binary mask. Scale bars, 1.5 μm.

Before we apply our analysis to the detection of very small proteins, we investigate its performance on a bovine serum albumin (BSA) sample, which, with a molecular mass of approximately 66 kDa, is one of the smallest proteins that can be detected with existing techniques. In Fig. 2a we show an example of the raw image recorded on the iSCAT camera, and Fig. 2b shows a single protein from that measurement after a typical DRA analysis on 1,500 neighboring frames. Although a small protein is successfully detected, the image also shows background fluctuations that are not fully eliminated by the existing algorithm, possibly due to various electronic, mechanical or fluidic sources of noise (Supplementary Information, Section 1 and ref. ^14^).

For our current discussion, it suffices to consider the residual signal fluctuations as ‘noise’ in the recognition of the particle contrast, which acts as the ‘signal’. Thus, the problem can be reduced to the challenge of deciphering image attributes at a given signal-to-noise ratio (SNR). In our set-up, proteins with a molecular mass of 40 kDa, which is the lowest that has been reported in the literature^11^, have an SNR of ~3, whereby the noise level is defined as the root mean square (RMS) of the residual background fluctuations. Here, it is important to note that the resulting speckle noise is not white because the spatial variations of the background are governed by the same instrument response function that determines the system PSF. This structured background makes it particularly difficult to identify the signal^18^. In this work, we show that the application of machine learning algorithms enables us to detect proteins as small as 9 kDa, corresponding to an SNR of ~1.4 in our set-up.

To improve the robustness of the results, we labeled the proteins under study with ATTO 647 dye molecules with a negligible molecular weight of approximately 0.7 kDa and negligible extinction coefficient at the iSCAT illumination wavelength, so that we can monitor them via the accompanying TIRF detection (Fig. 2c). To check the purity of the protein samples after labeling, we ran a gel electrophoresis (sodium dodecylsulfate–polyacrylamide gel electrophoresis) (Supplementary Information, Section 2). We note that variations in the number of fluorophores per protein do not disturb the study because we aim to identify only the protein. We found that co-illumination of the red and blue laser beams led to fast photobleaching, preventing us from performing simultaneous iSCAT and TIRF measurements (Supplementary Information, Section 10). We, thus, interlaced the two recording periods with typical repetition cycles of 30 s.

We now examine the same measurements using anomaly detection. Figure 2d presents the location of the resulting anomalous pixels for all frames that were used to detect the protein under discussion by using a user-defined feature matrix (Supplementary Fig. 8). To suppress false-positive events, we apply a morphological operation to eliminate unconnected anomalous pixels in each frame. In the case of the data in Fig. 2d, the morphological operation considered anomalous pixels that were accompanied by at least one more neighboring pixel (Supplementary Table 1). Next, the image in Fig. 2d is convolved with a Gaussian function that fits our experimental PSF, corresponding to half-width at half maximum of 2.5 pixels. We then implement a binary mask with a radius of 5 pixels about the center of mass of the resulting distribution to restrict a detection region for one landing event (Fig. 2e). In other words, two detection events are counted as such only if their binary masks do not overlap. A comparison with the conventional DRA and TIRF measurements (Fig. 2b,c) shows very good agreement with the outcome of anomaly detection based on user-defined criteria.

Figure 2f shows the result of anomaly detection based on the DNN approach for the protein landing event of Fig. 2b. It can be seen that as opposed to the user-defined scenario in Fig. 2d, the DNN approach can effectively isolate the entirety of a PSF in each frame, significantly increasing the detection yield. We note that to eliminate artifacts near the borders and corners of a frame, we considered only the data inside a circular mask of radius *R* = 33 pixels.

**Fig. 3:**
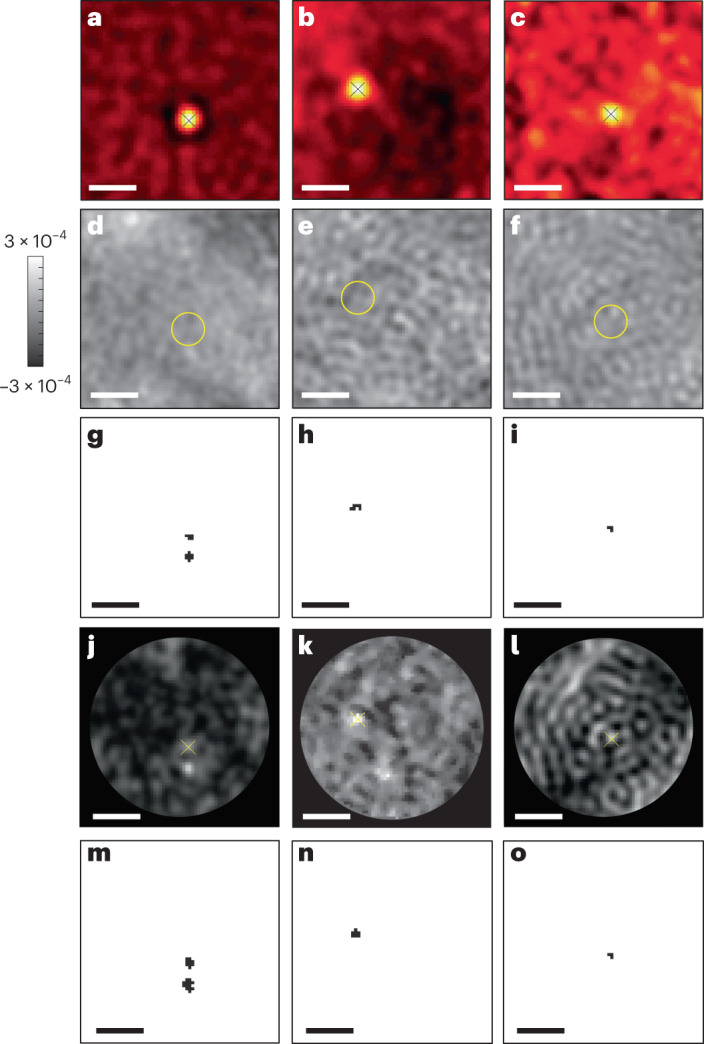
Detection of very small proteins with molecular mass of 21 kDa (left column), 18 kDa (middle column) and 9 kDa (right column). **a**–**c**, Single frames of raw TIRF videos associated with the landing of 21 kDa (**a**), 18 kDa (**b**) and 9 kDa (**c**) proteins. Crosses illustrate the centers of the deduced landing events. **d**–**f**, Results of a standard DRA analysis on 4,000 (**d**), 8,000 (**e**) and 8,000 (**f**) neighboring frames. Yellow circles are placed at the locations of protein landings which were localized in the corresponding TIRF channel and the color bar shows the iSCAT contrast, *C*. **g**–**i**, The outcome of iForest classification for a user-defined anomaly detection. **j**–**l**, Probability maps obtained from a DNN analysis. **m**–**o**, Results of iForest classification based on probability maps in **j**–**l**. Images in **g**–**o** are obtained for the same corresponding single DRA frames in **d**–**f**. Scale bars, 1.5 μm.

Having established the principle of our new methodology, we now showcase its performance by measuring proteins not previously detectable. Figure 3a–c shows examples of three TIRF images, which confirm the presence of proteins with a molecular mass of 21, 18 and 9 kDa, respectively. In Fig. 3d–f we present the corresponding DRA-treated images. To guide the eye, we placed circles at the locations of protein landing events as determined from the centers of the PSFs in their corresponding TIRF images. Distinguishing the protein PSF from the speckle-like background appears not to be within reach in any of the cases. Remarkably, however, the data in Fig. 3g–i show that anomaly detection based on user-defined features can identify the protein landing events. The success of this procedure can be traced to the fact that by combining temporal and spatial features in the feature matrix, the algorithm imposes simultaneous temporal and spatial restrictions that distinguish true landing events from other uncorrelated temporal and spatial fluctuations (Supplementary Information, Section 5). Figure 3j–l shows the probability maps of the events obtained from an unsupervised DNN analysis, and Fig. 3m–o plots the corresponding outcome of iForest classification. Both the user-defined and the DNN approaches succeed in detecting the protein events in the data presented in Fig. 3. The advantage of the latter method is, however, that it does not rely on optimal choices in the feature matrix. We compare the performances of the two methods in more detail in the Supplementary Information.

**Fig. 4:**
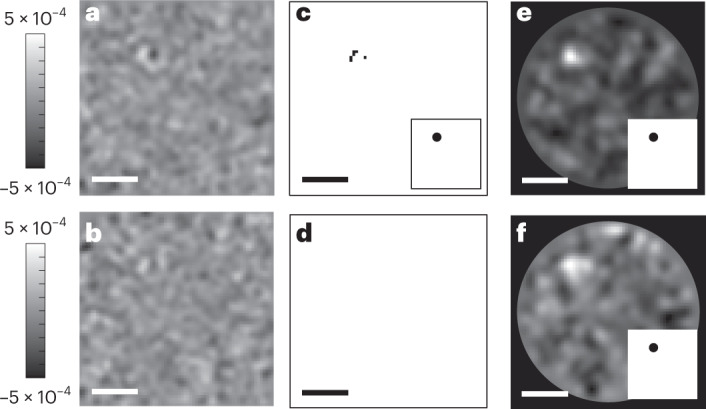
User-defined versus DNN performance at different SNR. **a**,**b**, Outcome of DRA for averaging window sizes of 750 (**a**) and 250 (**b**) frames applied to the data in Fig. 2. The color bars show the iSCAT contrast, *C*. **c**,**d**, Outcome of anomaly detection based on a user-defined feature bank for the DRA window sizes of 750 (**c**) and 250 (**d**) frames. **e**,**f**, Probability maps based on the DNN approach for the DRA window sizes of 750 (**e**) and 250 (**f**) frames. Insets in **c**,**e**,**f** show the corresponding binary masks. Scale bars, 1.5 μm.

To elucidate the advantage of the DNN further, we synthetically lowered the SNR of the landing event discussed in Fig. 2 by reducing the DRA window size. Figure 4a,b shows the outcome of two DRA averaging window sizes of 750 and 250 frames, respectively. As shown in Fig. 4d, the user-defined approach is not able to detect the protein with the same feature matrix criteria as before. Figure 4e,f, however, shows that the DNN approach remains successful.

**Fig. 5:**
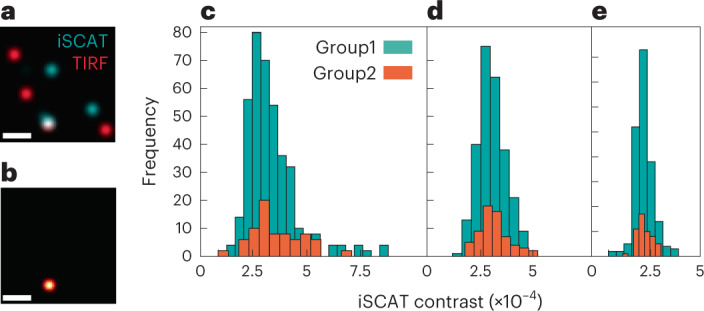
Comparison of the coincidence yields between iSCAT and TIRF. **a**, Overlay of landing events for a protein sample at 21 kDa recorded on the TIRF (red) and iSCAT (blue) channels during a period of 20 s. **b**, Correlation map between the iSCAT and TIRF channels. **c**–**e**, Histograms of the binding events for the 21 kDa (**c**), 18 kDa (**d**) and 9 kDa (**e**) protein samples. Group 1 shows the contrast value of the localized proteins resulting from DNN-based anomaly detection. Group 2 represents the binding events that appeared in both the TIRF and iSCAT channels. The data in **c**–**e** represent a collection of 322 (**c**), 263 (**d**) and 201 (**e**) single-protein data points from 1.93 (**c**), 4.75 (**d**) and 2.49 (**e**) million frames (Supplementary Table 3). Scale bars, 1.5 μm. Source data

We have presented several cases in which iSCAT detection of protein landing events was confirmed by TIRF images. The modulation of the iSCAT contrast in the speckle-like background, however, may cause false-positive events or mask a true event. Similarly, landing events might be absent in the TIRF channel, for example, due to photobleaching or imperfect labeling. Consequently, the yield in obtaining a one-to-one correspondence between the TIRF and the iSCAT data is low in our interlaced measurements (Supplementary Information, Section 10). One such example is shown in Fig. 3g,j,m, in which anomaly detection detects two proteins while TIRF finds only one of them. Figure 5a shows another example of several events captured in the iSCAT and TIRF channels recorded within 20 s. In Fig. 5b we show the coincidence map of the two signals obtained by constructing the pixel-wise product of the localized events. We note, however, that the average rate of landing events was comparable in the iSCAT and TIRF channels with 0.2–2 proteins per second and 1–5 proteins per second, respectively, showing that we do not over-count in the iSCAT channel. Furthermore, by performing simulations, we estimated the false-positive signals in our algorithms to be less than approximately 10% (Supplementary Fig. 11). In practice, one can choose to apply more stringent morphological operations on the DNN output to reduce the false-positive events at the cost of the detection yield (Supplementary Table 3). For instance, we included only events with at least three connected pixels in each frame for the 9 kDa data to minimize the chances of counting unwanted events (Supplementary Table 1).

### Protein mass photometry

The task of the anomaly detection algorithms (user-defined or DNN) discussed above is to identify protein landing events. Once the PSFs of individual particles have been localized, their iSCAT contrasts can be extracted as in previous reports to arrive at their mass information^14^ (Supplementary Fig. 3). In brief, the hot region identified by anomaly detection is searched using difference of Gaussian to localize the PSF of the protein (Supplementary Fig. 8g). We then extract the temporal value of the localized PSF center intensity directly from DRA to form a V-shaped landing trajectory (Supplementary Fig. 8c). The sides of the V-shaped trace are fitted with two lines and the intersect is used to assign the base line, which is then used to determine a contrast^14^.

The blue histograms in Fig. 5c–e show the distribution of the iSCAT contrasts obtained from 21, 18 and 9 kDa protein samples, respectively, following the full DNN-based anomaly detection algorithm. In addition, the orange histograms in Fig. 5c–e show the spread of the contrasts obtained for the iSCAT events that coincided with an event detected in the TIRF channel. We find that although the yield is lower for coincidences, the main modes of the histograms are very well aligned. We note that the distribution towards higher contrasts can be attributed to small populations of oligomeric states of the protein, protein aggregates, or sample impurities^12^. The Gaussian mixture model^28^ was used to identify the underlying subpopulations^14,29^.

**Fig. 6:**
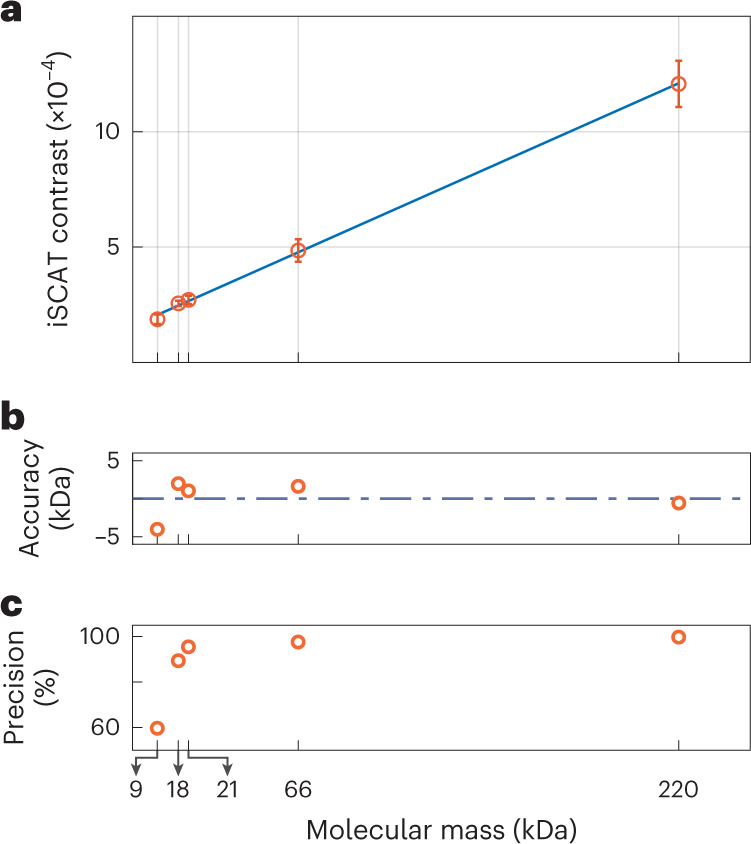
Protein contrast library. **a**, Five different samples, ranging from 9 to 220 kDa were measured and compiled to form the library. The iSCAT contrast scales linearly with the nominal molecular mass of the protein sample. The error bars were deduced by bootstrapping the data (see text). **b**, The distance of the points from the fitted linear model was calculated in terms of molecular mass to produce a measure for accuracy. The horizontal dashed line indicates zero. **c**, The precision in assigning the mean of a histogram associated with each data point in **a** (See Fig. 5c–e). Source data

The contrast of the main histogram mode was estimated using maximum likelihood estimation, analogous to the procedure in localization microscopy^30^. We then used bootstrapping to estimate the confidence interval in this assignment (sampling cycles >1,000). The deduced contrast can be related to mass if one assumes a common density and refractive index for proteins^8,12,14^. Because the parameters *r* and *s* (Eq. (1)) can vary between individual iSCAT set-ups, one needs to establish a calibration ladder, much in the spirit of the read-out procedure in gel electrophoresis. Figure 6a presents such a library, which contains the data from protein samples with nominal molecular mass of 220, 66, 21, 18 and 9 kDa. The error bars represent the precision in each assignment, and the line shows the result of a linear fit to the data.

In Fig. 6b we plot the accuracy (in units of kDa), determined as the difference between the measured mean value and the quantity suggested by the fit. Figure 6c presents the percentage precision. It is evident that both accuracy and precision become less robust for the smallest protein size. We also note a slight offset at the intercept of the linear fit on the vertical axis. We attribute this to the fact that the background fluctuations cannot be fully eliminated, thus, affecting the base line and the contrast value^14^. Nevertheless, the linear model in Fig. 6a has an RMS deviation of 1.0 × 10^−5^, which is one of the lowest values reported for such protein libraries^12,29^.

## Discussion and outlook

In 2014 iSCAT was successful in the label-free detection of single 500 kDa (myosin 5a)^31^ and 66 kDa (BSA)^8^ proteins. Since then, the sensitivity limit has been somewhat improved to 55 kDa^12^ and approximately 40 kDa^11^, whereby the application of a spatial mask in the Fourier plane was considered to be instrumental for favoring the scattered signal^12,32,33^. In our current work, we use an anomaly detection machine learning algorithm to substantially push the sensitivity limit to proteins as small as 9 kDa. Moreover, we achieve this without using a spatial mask.

Label-free and real-time analysis of small proteins is very promising for ultrasensitive diagnostics of disease markers such as interleukins or other cytokines in bodily fluids^34^. In addition, a range of fundamental studies such as assembly of biological nanostructures^35^, cell secretion^36,37^ and protein aggregation^38^ would greatly benefit from this methodology. iSCAT detection of biomolecules can be further advanced through improvements in physical measurements, for example, by using CMOS (complementary metal oxide semiconductor) cameras with larger well capacity and lower dark noise, or using a higher quality substrate surface to lower the iSCAT background. The methodology presented in this work also holds promise for efforts in cryogenic electron microscopy and fluorescence microscopy with low SNR. As machine learning approaches become more established in microscopy^18–21,23,39^, one can expect further advances in the computational analysis of label-free sensing. A first measure, for example, could involve replacing iForest with an end-to-end DNN^40^.

## Methods

### Protein sample preparation and labeling

All proteins used in this study are commercially available in a highly pure quality. Human plasma fibronectin (220 kDa) was purchased from Sigma Aldrich (cat. no. FC010). UltraPure BSA was purchased from Life Technologies (cat. no. AM2616). The structure of BSA corresponds to 66 kDa. The product used in this study was specified by the manufacturer at 67–68 kDa. Recombinant protein G (21 kDa) was purchased from Fisher Scientific (cat. no. 21193). Recombinant *Escherichia coli* Skp protein (18 kDa) and recombinant human interleukin (IL)-8 protein (9 kDa) were purchased from Abcam (cat. nos. ab97397 and ab9631, respectively). Proteins were diluted or buffer exchanged (desalted) into labeling buffer containing 50 mM HEPES and 25 mM KCl (pH 7.8), prior to the labeling reaction, using a 7K MWCO (molecular weight cut-off) Zeba desalting column (ThermoFisher, cat. no. 89882). Proteins were unspecifically labeled via their exposed primary amines using the ATTO 647 fluorophore containing the reactive group NHS (*N*-hydroxysuccinimidyl) ester (cat. no. 18373-1MG-F, Sigma Aldrich). Proteins were mixed with dyes at a ratio of 1:1 for 2 h at room temperature, and then desalted from the excess of dye using the same desalting columns. Proteins were further filtered using a 100 nm syringe filter (Whatmann Anotop 10, cat. no. WHA68091002, Sigma Aldrich). The labeling efficiency was then estimated using an absorption spectrometer (Nanodrop 2000, ThermoFisher). The labeling efficiency ranged between 40% and 80% for different protein samples. SDS–PAGE was used to assess protein purity, labeling and the approximate molecular weight (Supplementary Information). Based on the manufacturer information, most of these proteins are found in their monomeric states. In the case of Skp protein it can form a trimer assembly, however at the concentration of our measurements (~10 nM) it is mainly in the monomeric state^41^. To establish the protein ladder we read the contrast for the main (lowest) mode of the iSCAT histogram. We note that if proteins do form large assemblies, their larger iSCAT contrasts become noticeable in our experiments.

### Coverglass functionalization

To prepare the surface of the coverglass for protein binding, it was sonicated in isopropyl alcohol and ethanol for 5 min each, followed by 10 min of oxygen plasma. The sample was then mounted and left to stabilize for a few hours.

### Protein injection and data acquisition

Each labeled protein sample was diluted down to approximately 10 nM in concentration, and 10 μl was manually injected by micropipetting on top of the iSCAT field of view. This is then immediately followed by starting the iSCAT camera data acquisition, which triggers the blue iSCAT laser. After approximately 20 s of data acquisition, the blue laser is switched off and the red laser (TIRF channel) is switched on for 10 s. This is then followed by several cycles of interlaced iSCAT and TIRF data acquisition, to reach a satisfactory data volume for meaningful statistics. Depending on the protein size, the iSCAT camera was set to run at 5–15 kHz at an exposure time of 20 μs.

### Optical set-up

A continuous-wave laser centered at 445 nm (iBeam smart, Toptica) is collimated and focused onto the back focal plane of an oil-immersion microscope objective (*α* Plan-Apochromat ×100, NA 1.46, Zeiss). A coverglass is positioned at the focus of the microscope objective using a piezo positioner (Nano-LPQ, Mad City Labs). The iSCAT field is imaged using a scientific CMOS camera (MV1-D1024E-160-CL, Photonfocus).

TIRF illumination was done with a laser beam at 631 nm, which was directed into the iSCAT pathway via a dichroic mirror (D1, Chroma ZT647rdc-UF3) mounted on a translation stage and a second dichroic mirror (D2, Chroma T480spxxr-UF3). The fluorescence signal was collected via the same microscope objective that was used for the iSCAT measurements. D2 separated the fluorescence from the iSCAT path and transmitted it through D1 onto a CCD (charge-coupled device) camera (Hamamatsu Orca Flash). Here, we also used a band pass filter (ET700/75) in front of the camera (S1).

### Statistics and reproducibility

Single-protein sensitivity is achieved only when thousands of frames are averaged in the analysis procedure described here. Each detection event in Figs. 2–4 is by definition a single-molecule event and as such is not reproducible. However, in a given video containing millions of frames, hundreds of single-protein events are registered, which are nominally equivalent. The histograms in Fig. 5c–e are formed by considering all such individual recordings. The data points in Fig. 6a are read from such histograms.

### Reporting summary

Further information on research design is available in the Nature Portfolio Reporting Summary linked to this article.

## Online content

Any methods, additional references, Nature Portfolio reporting summaries, source data, extended data, supplementary information, acknowledgements, peer review information; details of author contributions and competing interests; and statements of data and code availability are available at 10.1038/s41592-023-01778-2.

## Supplementary information

Supplementary Information Supplementary Figs. 1–19, Supplementary Tables 1–4.

Reporting Summary

## Data Availability

The data are available upon reasonable request. Source data are provided with this paper.

## Source data

Source Data Fig. 5c iSCAT contrast of binding events for a protein mass of 21kDa detected only via iSCAT (group 1) and also in TIRF (group 2) as one zip file including a *.mat data file with Matlab script to plot the histograms.
Source Data Fig. 5d iSCAT contrast of binding events for a protein mass of 18kDa detected only via iSCAT (group 1) and also in TIRF (group 2) as one zip file including a *.mat data file with Matlab script to plot the histograms.
Source Data Fig. 5e iSCAT contrast of binding events for a protein mass of 9kDa detected only via iSCAT (group 1) and also in TIRF (group 2) as one zip file including a *.mat data file with Matlab script to plot the histograms.
Source Data Fig. 6a A MATLAB script which plots the iSCAT contrast of different protein samples vs. the molecular mass in units of kDa.

## Code availability

The scripts reported in this paper have been deposited at PiSCAT^42^: https://github.com/SandoghdarLab.

## References

[1] Hong M, Zhang Y, Hu F (2012). Membrane protein structure and dynamics from NMR spectroscopy. Annu. Rev. Phys. Chem..

[2] Zhu Z, Lu JJ, Liu S (2012). Protein separation by capillary gel electrophoresis: a review. Anal. Chim. Acta.

[3] Bai X-C, McMullan G, Scheres SH (2015). How cryo-EM is revolutionizing structural biology. Trends Biochem. Sci..

[4] Sauer M, Heilemann M (2017). Single-molecule localization microscopy in eukaryotes. Chem. Rev..

[5] Kaushik A (2019). Advances in nanosensors for biological and environmental analysis: book review. Biosensors.

[6] Sandoghdar V (2020). Nano-optics in 2020 ± 20. Nano Lett..

[7] Kukura P, Celebrano M, Renn A, Sandoghdar V (2010). Single-molecule sensitivity in optical absorption at room temperature. J. Phys. Chem. Lett..

[8] Piliarik M, Sandoghdar V (2014). Direct optical sensing of single unlabelled proteins and super-resolution imaging of their binding sites. Nat. Commun..

[9] Lindfors K, Kalkbrenner T, Stoller P, Sandoghdar V (2004). Detection and spectroscopy of gold nanoparticles using supercontinuum white light confocal microscopy. Phys. Rev. Lett..

[10] Taylor RW, Sandoghdar V (2019). Interferometric scattering microscopy: seeing single nanoparticles and molecules via Rayleigh scattering. Nano Lett..

[11] Priest L, Peters JS, Kukura P (2021). Scattering-based light microscopy: from metal nanoparticles to single proteins. Chem. Rev..

[12] Young G (2018). Quantitative mass imaging of single biological macromolecules. Science.

[13] Taylor, R. W. & Sandoghdar, V. in *Label-Free Super-Resolution Microscopy* (ed. Astratov, V.) Ch. 2 (Springer International Publishing, 2019).

[14] Dastjerdi HM (2021). Optimized analysis for sensitive detection and analysis of single proteins via interferometric scattering microscopy. J. Phys. D. Appl. Phys..

[15] Pang G, Shen C, Cao L, Hengel AVD (2021). Deep learning for anomaly detection: a review. ACM Comput. Surv..

[16] Cheng C-Y, Hsieh C-L (2017). Background estimation and correction for high-precision localization microscopy. ACS Photonics.

[17] Spindler S, Sibold J, Gholami Mahmoodabadi R, Steinem C, Sandoghdar V (2018). High-speed microscopy of diffusion in pore-spanning lipid membranes. Nano Lett..

[18] Möckl L, Roy AR, Petrov PN, Moerner WE (2019). Accurate and rapid background estimation in single-molecule localization microscopy using the deep neural network BGnet. Proc. Natl Acad. Sci. USA.

[19] Wang F, Henninen TR, Keller D, Erni R (2020). Noise2atom: unsupervised denoising for scanning transmission electron microscopy images. Appl. Microsc..

[20] Speiser A (2021). Deep learning enables fast and dense single-molecule localization with high accuracy. Nat. Methods.

[21] Špačková B (2022). Label-free nanofluidic scattering microscopy of size and mass of single diffusing molecules and nanoparticles. Nat. Methods.

[22] Tassano, M., Delon, J. & Veit, T. Fastdvdnet: towards real-time deep video denoising without flow estimation. In *Proc. 2020 IEEE/CVF Conference on Computer Vision and Pattern Recognition*, 1354–1363 (IEEE, 2020).

[23] Sheth, D. Y. et al. Unsupervised deep video denoising. In *Proc. 2021 IEEE/CVF International Conference on Computer Vision (ICCV)*, 1759–1768 (IEEE, 2021).

[24] Ting, K., Liu, F. & Zhou, Z. Isolation forest. In *Proc. 8th IEEE International Conference on Data Mining**(ICDM)*, 413–422 (IEEE, 2008).

[25] Midtvedt B (2021). Quantitative digital microscopy with deep learning. Appl. Phys. Rev..

[26] Xu Y, Wu T, Gao F, Charlton JR, Bennett KM (2020). Improved small blob detection in 3D images using jointly constrained deep learning and Hessian analysis. Sci. Rep..

[27] Chandola V, Banerjee A, Kumar V (2009). Anomaly detection: a survey. ACM Comput. Surv..

[28] McLachlan, G. J. & Peel, D. *Finite Mixture Models* (John Wiley & Sons, 2004).

[29] Sonn-Segev A (2020). Quantifying the heterogeneity of macromolecular machines by mass photometry. Nat. Commun..

[30] Mortensen KI, Churchman LS, Spudich JA, Flyvbjerg H (2010). Optimized localization analysis for single-molecule tracking and super-resolution microscopy. Nat. Methods.

[31] Ortega Arroyo J (2014). Label-free, all-optical detection, imaging, and tracking of a single protein. Nano Lett..

[32] Cole D, Young G, Weigel A, Sebesta A, Kukura P (2017). Label-free single-molecule imaging with numerical-aperture-shaped interferometric scattering microscopy. ACS Photonics.

[33] Liebel M, Hugall JT, van Hulst NF (2017). Ultrasensitive label-free nanosensing and high-speed tracking of single proteins. Nano Lett..

[34] Seruga B, Zhang H, Bernstein LJ, Tannock IF (2008). Cytokines and their relationship to the symptoms and outcome of cancer. Nat. Rev. Cancer..

[35] Garmann RF, Goldfain AM, Manoharan VN (2019). Measurements of the self-assembly kinetics of individual viral capsids around their RNA genome. Proc. Natl Acad. Sci. USA.

[36] Zhang M, Schekman R (2013). Unconventional secretion, unconventional solutions. Science.

[37] McDonald MP (2018). Visualizing single-cell secretion dynamics with single-protein sensitivity. Nano Lett..

[38] Ross, C. A. & Poirier, M. A. Protein aggregation and neurodegenerative disease. *Nat. Med.* **10** (Suppl.), S10–S17 (2004). 10.1038/nm1066 15272267

[39] Li Y, Xue Y, Tian L (2018). Deep speckle correlation: a deep learning approach toward scalable imaging through scattering media. Optica.

[40] Wang D, Wang X, Lv S (2019). An overview of end-to-end automatic speech recognition. Symmetry.

[41] Pan S, Yang C, Zhao XS (2020). Affinity of skp to OmpC revealed by single-molecule detection. Sci. Rep..

[42] Dastjerdi HM, Mahmoodabadi RG, Bär M, Sandoghdar V, Köstler H (2022). PiSCAT: a Python package for interferometric scattering microscopy. J. Open Source Softw..

