# Supplementary material for: Self-supervised machine learning pushes the sensitivity limit in label-free detection of single proteins below 10 kDa
Source: Nat Methods. 2023 Feb 27;20(3):442–7. doi: 10.1038/s41592-023-01778-2 (PMC9998267; doi:10.1038/s41592-023-01778-2)
Supplement: Supplementary file 1 — Supplementary Figs. 1–19, Supplementary Tables 1–4. [file 41592_2023_1778_MOESM1_ESM.pdf]

nature methods

Article

https://doi.org/10.1038/s41592-023-01778-2

Self-supervised machine learning pushes the sensitivity limit in label-free detection of single proteins below 10 kDa

In the format provided by the authors and unedited

Self-supervised machine learning pushes the sensitivity limit in label-free detection of single proteins below 10 kDa

Mahyar Dahmardeh[1,2,†], Houman Mirzaalian Dastjerdi[1,2,3,†], Hisham Mazal[1,2], Harald Köstler[3,4], and Vahid Sandoghdar[1,2,5,*]

[1]Max Planck Institute for the Science of Light, 91058 Erlangen, Germany.
[2]Max-Planck-Zentrum für Physik und Medizin, 91058 Erlangen, Germany.
[3]Department of Computer Science, Friedrich-Alexander-Universität Erlangen-Nürnberg, 91058 Erlangen, Germany.
[4]Erlangen National High Performance Computing Center (NHR@FAU).
[5]Department of Physics, Friedrich-Alexander-Universität Erlangen-Nürnberg, 91058 Erlangen, Germany.
[*]To whom correspondence should be addressed. E-mail: vahid.sandoghdar@mpl.mpg.de
[†]these authors contributed equally to this work

1 Stability and noise analysis

1.1 Testing the stability of the optical setup

To monitor the jitter and drift of the optical setup, we etched a standard coverglass to produce indentation pockets with diameter of 300 nm and depth of 50 nm. Supplementary Fig. 1a shows the PSF of an etched pocket in iSCAT. A series of frames were then acquired to track the etched pocket (exposure time of 10 μs and frame rate of 100 Hz). Supplementary Figs. 1b and 1c show the localized traces in x and y directions as a function of the camera pixels. The etched pocket has a movement of about 5 and 8 Angstroms in x and y, respectively, over the course of 30 minutes. This is much longer than a typical iSCAT protein measurement ($\sim$20 seconds). This confirms the very high stability of the setup.

1.2 Background contrast fluctuation and SNR

In order to determine the best signal-to-noise ratio (SNR), a series of control measurements were made with a blank buffer over the same coverglass prior to injection of proteins. We then performed differential rolling average (DRA) over the image and varied the batch size over the acquired data. We evaluated the spatial fluctuations of the iSCAT signal over the image to establish a noise plot. Supplementary Fig. 1d depicts the theoretically expected shot noise (red line) and experimentally obtained fluctuations (blue line) as a function of the averaging batch size for a bare coverglass. Supplementary Fig. 1e displays the same quantity after proteins were injected. It is seen that at some point the blue curve starts to deviate form the red line. In other words, increasing the integration time does not improve the SNR beyond this point and instead smears the signal of interest. We thus used this duration to process all proceeding data.

2 Protein sample purity

In order to verify the molecular mass and purity of proteins labeled with ATTO647-NHS in this study, we ran them through SDS-PAGE (4–15% Mini-PROTEAN TGX Precast Protein Gels). Supplementary Fig. 2 shows the results. Lanes#1-5 show the human plasma fibronectin (220 kDa), bovine serum albomine ($\approx$ 66 kDa), recombinant protein G (21 kDa), recombinant E. Coli Skp protein (18 kDa) and recombinant human IL-8 protein (9 kDa) respectively. Lane L shows the protein standard ladder (PM2500). The gels show one solid band, indicating the purity and homogeneity of the samples.

3 Standard analysis algorithm

The standard analysis of iSCAT protein detection and mass photometry is discussed in detail in Ref[1]. Here, we give a brief summary of the concept. After injection of proteins, a series of iSCAT data is acquired. The raw iSCAT images are fed to the analysis algorithm for detection and visualization of proteins (see Supplementary Fig. 3). As the first step, the images are

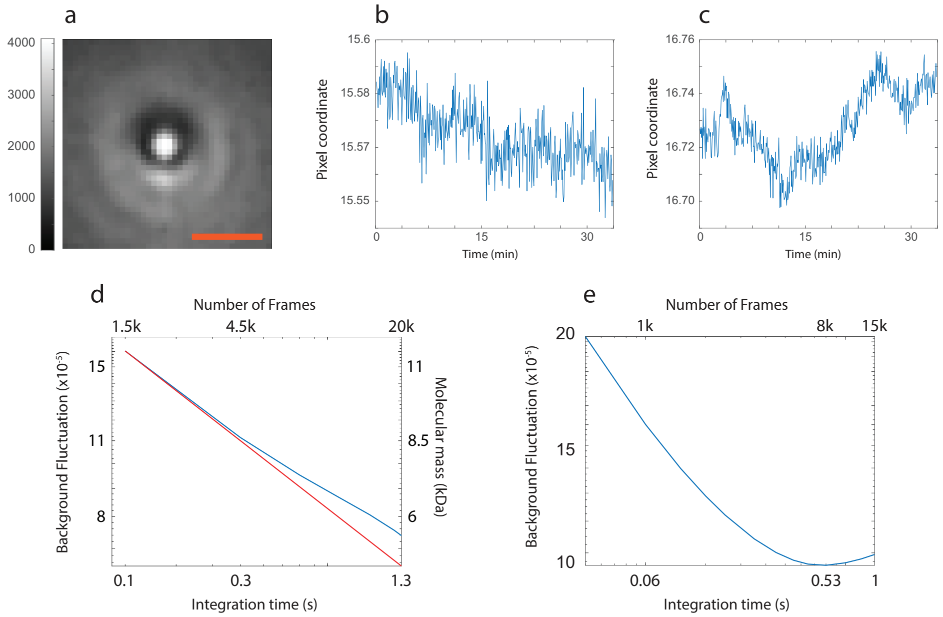

Supplementary Fig. 1. iSCAT setup stability. **a**, iPSF of an etched indentation pocket is tracked throughout time for measuring the mechanical stability of the setup. Scale bar corresponds to 1 μm. **b, c**, Temporal localization of the etched pocket in x, and y respectively, as a function of the camera pixel, corresponding to 5 and 8 Angstroms in our imaging scheme. **d**, Typical background fluctuations assessed on a bare coverglass that is thoroughly cleaned and imaged via iSCAT. A field of view of 72 by 72 pixels which corresponds to a square region of 6 μm in length over the coverglass is chosen, and a series of frames are acquired at 15 kHz. The frames are processed using differential rolling average (DRA) with varying batch sizes. Each pixel exhibits a certain temporal fluctuation. The variation is computed by taking the standard deviation of each pixel through out time. Subsequently, the mean value of all the standard deviations are calculated and assigned as the background fluctuation (noise) for the corresponding integration time. The blue line corresponds to the experimental values and the red line demonstrates the fluctuations, assuming the only source of noise to be the quantum fluctuations of the incident laser light. It can be seen that after an integration time of about 300 ms, the experimental values start to deviate from the theoretically expected shot noise. The y-axis also shows the equivalent molecular mass in kDa at SNR=1. **e**, Same as in (d) but after proteins were injected.

power normalized to account for laser power instabilities. Since, the field of view is chosen to be homogeneously illuminated, the values of all the pixels are simply added, and each pixel value is divided through the sum. Next, the images are corrected for fixed pattern noise (FPN). Then a differential rolling average (DRA) operation is applied to mainly mask the speckle-like background fluctuations to reveal the iSCAT PSFs of the landing proteins. This is then followed by a series of temporal and spatial filtering steps to ensure a reliable detection. For example, the spatial signature of a PSF is examined to ensure that it represents an actual protein and does not result from a neighboring speck-like background.

The filtered events are tracked through time and space to form a V-shaped trajectory[1], see Supplementary Fig. 8c. In the temporal domain, the tracked particle is required to be present in all frames; if it is absent in a few consecutive frames, that PSF is considered as noise. Given the high frame rate of acquisition (>5 kHz) and low diffusion rate of the protein sample ($\ll 100\ \mu m^2/s$), the localized particle is allowed to move within a neighborhood of only a few pixels in the spatial domain. Otherwise, it is regarded as noise. The iSCAT contrasts of the filtered PSFs are then used to form the V-shape profile. For each protein sample, a large number of PSFs are localized, tracked and assigned an iSCAT contrast value to form a histogram. The histogram is then dichotomized using Gaussian mixture model (GMM)[2], which is an unsupervised machine learning algorithm. The number of underlying populations are found using Akaike and Bayesian information criteria (AIC&BIC)[1,3,4]. GMM uses this number to effectively assort the histogram and assign a contrast for each sub-population. Using this information, one can also infer the mass heterogeneity of the sample. The main mode of the histogram is reported as the contrast of the protein. Supplementary Fig. 3 shows the pipeline flowchart.

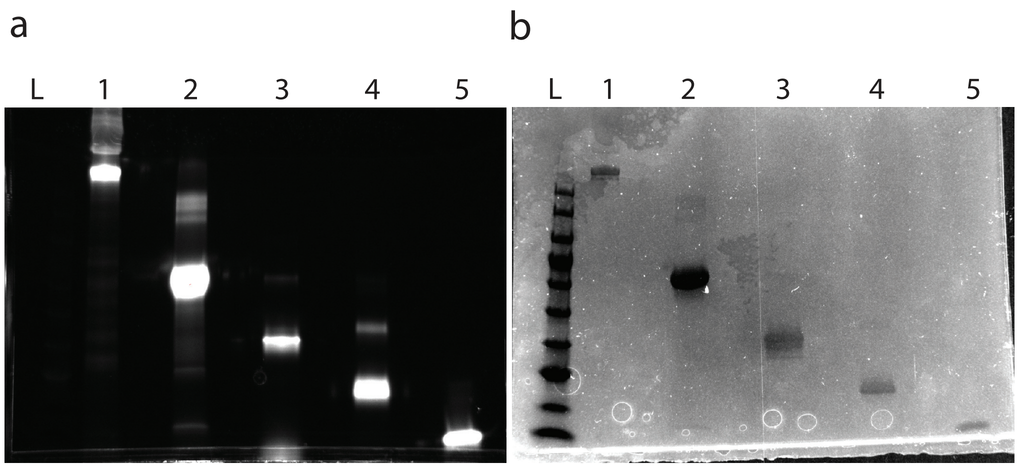

Supplementary Fig. 2. SDS gel electrophoresis (4–15% Mini-PROTEAN TGX Precast Protein Gels) of proteins labeled with ATTO647-NHS ran at 200 V for 35 min. **a**, The gel was imaged with a ChemiDoc XRS system at 647 nm. **b**, It was then stained with Coomassie Blue. Lane L is the protein ladder PM2500 (SMOBIO), lane 1 is Fbronectin (220 kDa), lane 2 is BSA ($\approx$ 66 kDa), lane 3 is Recombinant protein G (21 kDa), lane 4 is Recombinant E. Coli Skp protein (18 kDa) and Lane 5 is Recombinant human IL-8 (9 kDa). The gels show one major band, indicating a homogeneous and pure solution of the proteins.

4 Machine learning concepts used in this work

Our work exploits established machine learning concepts. We thus refer the interested reader to consult the vast literature in this area to learn about the subtleties and nuances of this very powerful method. In this section, we present a very brief non-technical overview of the central concepts used in our work.

4.1 Features

A crucial challenge in machine learning is to identify informative, discriminating, and independent characteristics or **features**[5]. Features should highlight parts of the information in a data set that are useful for classification, clustering, or regression. In our application, features can include the size and shape of the instrument response function (PSF) or temporal quantities such as the mean or standard deviation of a pixel intensity in the field of view. In the case of user-defined AD, a feature matrix is put together by the user based on physical and mathematical arguments. Indeed, feature extraction is also a vital task that is determined by humans in traditional methods.

4.2 Anomaly detection

Anomaly or outlier detection is a branch of artificial intelligence that distinguishes between normal and abnormal data and is usually used when no ground truth is available (unlabeled data)[6]. Approaches based on anomaly detection (AD) use extracted features to distinguish outliers from normal data (e.g., one-class SVM), where outliers are infrequent and have considerably different properties[7]. For this purpose, AD techniques use a higher dimensional space of features and a transform function (kernel) to classify data. One type of AD estimates the distribution model of the normal data and finds abnormalities based on the quality of fits to a model (see Supplementary Figs. 4a, b). A second type employs a features domain space to enhance the distance between the normal data and the outlier (see Supplementary Figs. 4c, d). In this work, we utilized the latter approach based on iForest (see below), which outperforms other techniques such as Robust Covariance, One-Class SVM, One-Class SVM (SGD), and Local Outlier Factor for our application. The feature matrix for identifying the isolated pixels in each frame (either acquired by user design or by DNN) is then fed to iForest.

4.2.1 Isolation Forest

Isolation Forest (iForest) is an unsupervised anomaly detection algorithm[8]. It isolates data by representing them in a set of decision tree-like structures. Each decision tree in the forest randomly chooses a set of features and attempts to isolate each data point with respect to the chosen set of features. By constructing many decision trees and repeating the same procedure for different sets of features, the data are clustered. Supplementary Figure 5a shows an example of a data set for features "X" and "Y". An exemplary tree in Supplementary Figure 5b shows that the first step (Depth level 1) looks at "X<13" and isolates the red point from the rest of the data. It then sets different decision values for "X", and "Y" until it isolates each data point at a certain depth level. Subsequently, for each data point an anomaly score is assigned according to the depth level at which it

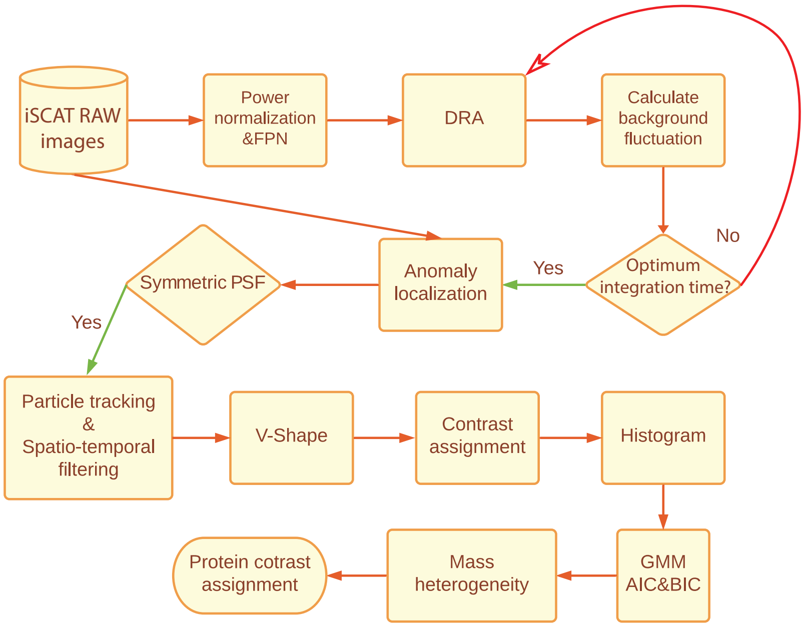

Supplementary Fig. 3. Analysis pipeline (see Ref. [1]). After acquisition of a series of frames (iSCAT data), they are fed to the analysis pipeline for visualizing and tracking the bindings of the protein samples. Each iSCAT video frame is power normalized by dividing the value of each pixel through the sum of the values from all pixels in that frame. This is followed by correcting for the fixed pattern noise of the camera and applying the DRA routine. Anomaly Detection is then applied to find the protein binding candid regions. iSCAT PSFs are localized within the candid regions and are tracked through time to form a temporal trajectory. Moreover, if the localized PSF is too asymmetric, it is considered as noise and removed. The tracked PSF has to be continuously present in a certain number of consecutive frames and not have moved more than a certain number of pixels during the tracking procedure (Spatio-temporal filtering). The iSCAT contrast of the temporal trajectory is further processed to make sure that it exhibits the V-shape signature of a landing event and if so, the extremum of it is registered as the contrast of that landing event. As the next step, all the registered contrasts are concatenated to form a histogram. The histogram is then dichotomized into sub-population using Gaussian mixture model in order to infer information regarding the mass heterogeneity of the sample. The acceptable number of the underlying population is given by Akaike's and Bayesian information criteria (AIC&BIC). According to the sub-populations, the main mode of the histogram is assigned as the iSCAT contrast of the analyte.

was isolated within the tree structure. iForest then constructs many decision trees, repeats the same procedure, and assigns an anomaly score to the data points within each tree structure, where different combinations of "X" and "Y" decision values are explored. The final **anomaly score** is calculated by taking the average score over all the decision trees (hence, the name iForest). The algorithm then defines the parameter **contamination** which can take values in the range 0-0.5 and is inversely proportional to the "anomaly score", i.e., the higher the anomaly score, the lower the contamination value. This quantity essentially reports on how anomalous the region is. For our iSCAT data we have used a contamination range of 0.002-0.003 to identify our PSFs from the speckle-like background.

4.3 Deep neural networks

Deep neural network (DNN) is a branch of machine learning that improves on classical machine learning tasks by automating feature extraction through a high degree of non-linearity[9]. To this end, DNNs contain a combination of linear and nonlinear components. During the training phase, trainable parameters are tweaked to extract the proper features in a manner that reduces the loss function defined for the DNN (error of DNN); DNN employs various optimizers to determine the appropriate weight settings for minimizing the loss function[9]. The output of a supervised DNN is compared with labelled data to tune trainable parameters, whereas a self-supervised DNN only compares the output with the input data. Unsupervised DNN is often predicated on some data assumption such as knowing the probability distribution of the output and attempting to maximize the likelihood. DNN is often classified by its architecture and layers, and the design varies based on the application.

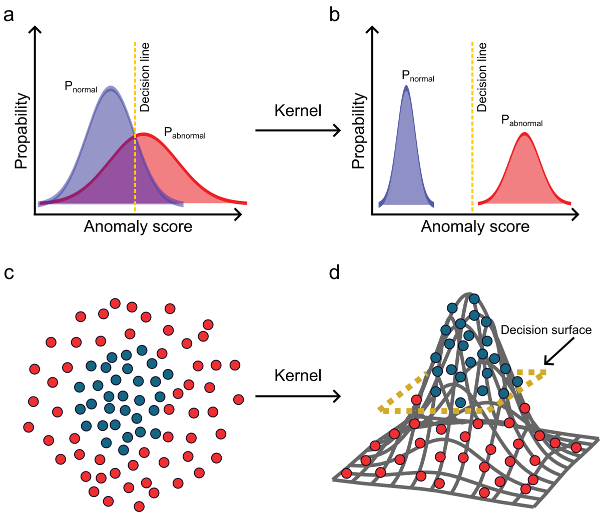

Supplementary Fig. 4. Two approaches in Anomaly detection. a) Probability distribution of normal (blue) and anomalous data (red). b) Anomaly uses a suitable kernel to separate the two probabilities using a linear decision boundary. c, d) The location of normal (blue) and anomalous data (red) in 2D-feature space (c) is transferred to a 3D-feature space (d) to allow for a linear separation.

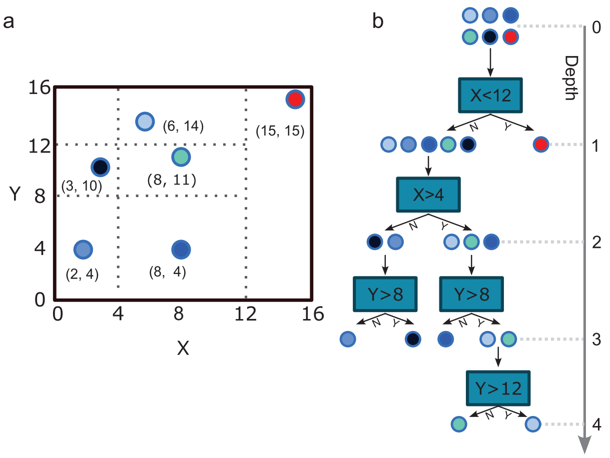

Supplementary Fig. 5. iForest pipeline. a) Distribution of six data points in a matrix of two features X and Y. b) Classification of the data points in (a) using a tree structure that makes an effort to divide up all points into distinct regions. In the initial tree depths, points that are isolated from the rest of the data are more likely to be labeled as anomalous.

4.3.1 U-Net

U-Net is a well-known DNN intended for image classification. The first generation of the U-Net architecture, which was created for image segmentation, is categorized as a convolution neural network and autoencoder[10]. Supplementary Fig. 6 shows the general architecture of a U-Net, which consists of five levels. Levels 1D, 2D and 3 extract the features from the data and encode them (thus the name encoder). Levels 3, 2U, and 1U reconstruct results from the previous levels. The levels are connected using various nonlinear/linear operations, namely, convolution, batch normalization (BN), ReLU, and PixelShuffel[10]. These operations are depicted using the colored arrows in Supplementary Fig. 6. The concept of each operation is as follows:

Convolution: The convolution layer works by convolving the previous layer with a matrix, which is initialized with random values that are tuned during the training process in order to decrease the system loss function. In the encoding process, the input data are downsampled. This can be done in different ways, including max-pooling, average-pooling, and stride[10].

BN: In the context of DNN, BN is utilized when two separate layers/levels are linked to normalize weights in a way that allows for comparing the aforementioned weights. BN significantly reduces the training time[11].

Pixel shuffling: Pixel shuffling is used for upsampling the data in order to minimize interpolation artifacts[12]. It should be noted that upsampling requires interpolating pixels which can end up in a number of image artifacts (e.g., checkerboard artifacts and

edge blur issues).
Skip connection: Skip connection between two levels is used to reduce degradation issues and vanishing gradients. Skip enhances the quality of feature extraction and image reconstruction[13].

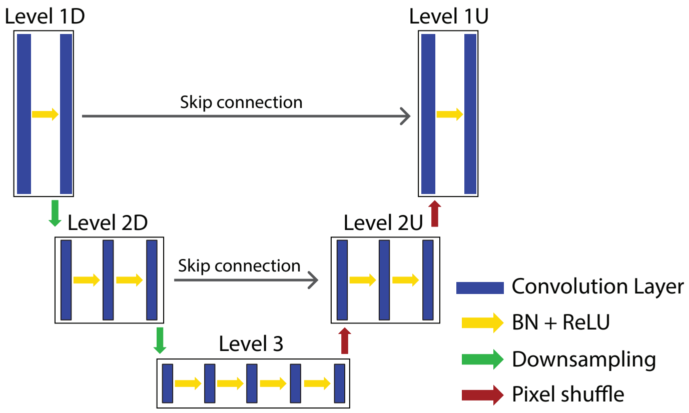

Supplementary Fig. 6. The U-Net design uses five levels of downsampling and upsampling. Levels 1D, 2D and 3 are used to encode the input data. Levels 3, 2U and 1U are employed for decoding the outputs of the previous layers/levels. Skip connection is used to connect two different levels. Each convolution layer is depicted using the blue bars. The arrows are color coded to depict the nonlinear/linear operations.

4.3.2 Towards Real-Time Deep Video Denoising Without Flow Estimation (FastDVDNet)

FastDVDNet is a DNN that is designed for video denoising[14]. As depicted by Supplementary Fig. 7, this DNN is developed based on four identical U-Nets and in two different stages, with an architecture similar to Supplementary Fig. 6. In level one, three U-Nets compare a target frame in the video with other nearby frames (two frames backwards and two frames forwards) to extract initial features. These features are fed to the 4th U-Net on level two to remove noise from the targert frame. DNN is trained by comparing the estimated frame to the input frames and using the mean square error (MSE) as the loss. In our case, DNN removes PSFs in test mode because their dynamics are faster than the speckle in the background. The resulting residual highlights the PSFs by calculating the difference between the input and estimated frames.

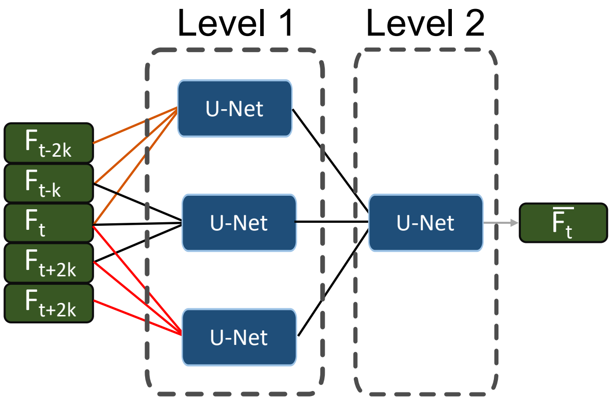

Supplementary Fig. 7. FastDVDNet architecture. Two levels are used for feature extraction. Four identical U-Nets are utilized to denoise target frame "t" by comparing two preceding frames of F_{t-k}, and F_{t-2k} and two proceeding frames of F_{t+k}, and F_{t+2k}.

5 Anomaly Detection based on user-defined feature matrix

iSCAT raw images and the outcome of DRA are used to extract the spatial and temporal features for AD. In order to evaluate the temporal features of a pixel at time t, two consecutive batches (with size r) before and after the frame at t are defined. In our case, we found that the mean values and standard deviations of a pixel throughout the time windows are effective statistical measures for finding an anomalous event. We denote $M1$, and $SD1$ as the mean value and the standard deviation of the pixel

during the first window, $M2$, and $SD2$ for the the second window, etc.. Moreover, in order to pin point the temporal changes in the nominal values of the pixel of interest, we probe the difference in the mean values ($M1-M2$), and the difference of the first ($M1-M12$) and the second mean ($M2-M12$) from the total mean value $M12$. The latter essentially corresponds to the bias of the pixel during the first and the second windows combined.

Spatial features are extracted after noise correction and DRA with batch size r. The spatial features are then obtained by running a Difference of Gaussian (DoG)[15] over the outcome of the DRA. Here, each video frame is convolved with two 2D-Gaussians of different sizes. The sigma of the larger one is chosen to be greater than the experimental PSF size, while the smaller one has a sigma equal to the size of our PSF (Supplementary Table 1). In other words, the larger Gaussian blurs the image and provides an estimate of the background, whereas the smaller one highlights the actual PSFs. The distinction between these two provides an enhanced image that improves the SNR of our candid PSFs. The parameters of DoG are tuned based on the experimental knowledge of the diffraction-limited spot of the iSCAT setup. The parameter list for DoG can be found in Supplementary table 1.

The temporal parameters of linking and tracking a PSF through time are given by Supplementary table 2. During the tracking process, a protein is allowed to move by a distance of 1 pixel. Also, it could happen that the algorithm is not able to localize the PSF for a short period of time due to noise. Thus we set a threshold ("memory") as the maximum number of consecutive frames that are allowed for the PSF to be missing before that landing event is discarded. We chose this threshold to be 25 to 50, depending on the frame rate and the batch size (see Supplementary table 2). The spatial and temporal features are then mixed to create a spatio-temporal feature map for each pixel. Next, the feature maps of all pixels at a given time stamp t are concatenated to form a feature matrix, as shown in Supplementary Fig 8h. Each row in a feature matrix represents a pixel and the columns are the spatio-temporal features. This is then fed to the Isolation Forest (iForest) for classification[16]. This algorithm randomly selects features and recursively partitions them into a tree structure by sub-sampling the data.

The outcome of iForest is a 1D vector representing normal and anomalous pixels as 0/1 values. This vector is reshaped back to a 2D matrix corresponding to a camera frame (Supplementary Figs. 8d-g), thus, establishing a binary mask. This mask is further distilled to eliminate false detection events by using a probability score ("morphological size"), which is derived from the total number of connected anomalous pixels in the binary mask (morphological operation in Supplementary Fig. 8). Doing so eliminates detected neighborhoods (a set of connected pixels) that are smaller than a certain size (see Supplementary table 1). This results in a binary mask that is then convolved with a Gaussian kernel with a sigma set to the size of our experimental iSCAT PSF and binarized. This in turn renders a set of hot regions that are searched using DoG for localization and further on, reading out the PSF contrast.

Mass (kDa)	Batch size		DoG		experimental PSF size (px)	Median filter (px)	Morphological size		iForest (contamination)		Stride
	Frames	ms	Sigma min (px)	Sigma Max (px)			User defined	DNN	User defined	DNN	DNN
9	8000	500	1.7	1.8	1.7	3	3	3	0.002	0.002	10
18	8000	500	1.7	1.8	1.7	3	3	3	0.002	0.002	10
21	4000	500	1.7	1.8	1.7	3	4	3	0.002	0.003	10
66	1500	300	1.7	1.8	1.7	3	2	2	0.002	0.002	10
220	1500	300	1.7	1.8	1.7	3	2	2	0.002	0.002	10

Supplementary Tabel 1. Hyper-parameter space used for the analysis pipeline. First column shows the molecular mass. Second column is the DRA batch size in terms of number of frames and the corresponding amount of time. Third column shows the parameters for tuning DoG. Fourth column shows the experimental PSF size in terms of camera pixel size. Fifth column is the size of the median kernel used to treat the iSCAT raw data in order to eliminate the dead pixels. Sixth column signifies the minimum number of connected pixels in order for the morphological operation not to eliminate them based on lack of connectivity. Seventh column shows the iForest thresholding scores for finding anomalies. The last column depicts the stride size for the U-Nets in DNN.

6 Anomaly Detection based on Deep Neural Network (DNN)

The iSCAT raw data are DRA treated and fed to a DNN as the training data. Here we have used FastDVDNet[17], a state-of-the-art structure based on U-Nets, for extracting spatial-temporal features from the data. The architecture combines four identical U-Nets. Each U-Net learns how to encode input data (down-sampling) to extract sufficient number of features that are used for decoder parts (up-sampling) to reconstruct target data ($\text{Frame}_{\text{predict}}$ in Supplementary Fig. 9) during the training steps. Firstly, each U-Net receives three input frames and employs a depth-wise convolution layer. Moreover, each U-Net has three down-sampling levels that are connected to the same level in up-sampling by a factor of two. Pixel-shuffle[18] is then used for the

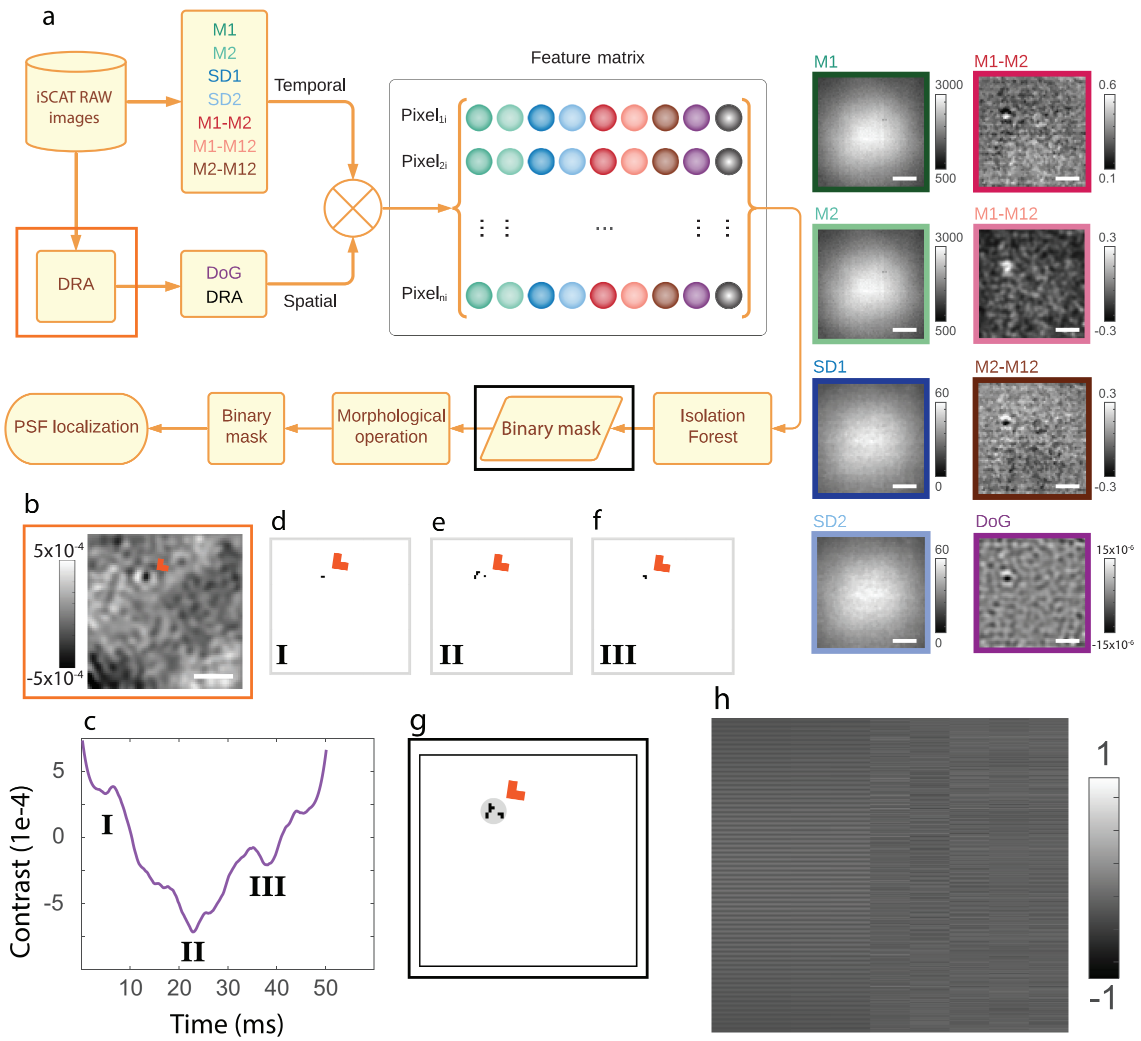

Supplementary Fig. 8. a, Anomaly Detection based on User-Defined Feature Matrix. Temporal features are extracted from the raw iSCAT data, and spatial features are extracted after noise correction and DRA. The DRA data are treated with a median filter with a kernel size of 3×3 to correct for dead pixels. Later on, two batches of frames with a length equal to the DRA batch size are defined and rolled over the raw iSCAT data. $M1$, $SD1$, and $M12$ denote the mean value and the standard deviation of the first rolling batch, and the mean value of the concatenated first and second batch. $M2$, and $SD2$ correspond to the same statistical measures but for the second batch. These parameters are employed to detect the temporal signature of a protein landing event. Moreover, the spatial features are formed based on the DoG and DRA treated iSCAT data. Sample frames for each feature are depicted on the right hand side of the figure and color coded accordingly. The spatio-temporal features are formed by conflating the spatial and temporal features. Based on this, a feature map for each pixel is formed and concatenated to form a feature matrix. iForest then classifies the events by randomly selecting features and recursively partitioning them into a tree structure. In this fashion, we render a binary mask that shows whether a pixel is normal or anomalous throughout time. The mask is further processed to eliminate false detections. A probability score (morphological size) is formed based on the total number of connected anomalous pixels in the probability mask (Morphological operation). This generates a binary mask which is then convolved with a Gaussian of the size of our PSF. The result is a set of hot regions that are processed using DoG for the final localization. **b**, The outcome of DRA-treated iSCAT data with the red arrow pointing towards a localized PSF of a BSA protein. **c**, The so-called V-shape of the landing event for the same protein sample. **d-f**, Sample masks of iForest corresponding to the time stamps marked in (**c**). **g**, Sum of all the masks, corresponding to the landing event in (**c**) with the hot region overlaid as a gray circle. **h**, The normalized feature matrix corresponding to the frame marked with (II) in (**c**). The matrix has 9 columns, depicting the spatio-temporal features and 5040 rows, corresponding to the total number of pixels in each features frame. Scale bars equate to 1.5 μm.

Mass (kDa)	Search range (Pixels)	Memory (frames)	Minimum Temporal length (frames)	Maximum Temporal length (frames)	Batch size (frames)
9	1	50	2400	16000	8000
18	1	50	3200	16000	8000
21	1	50	1600	8000	4000
66	1	25	1200	3000	1500
220	1	25	1200	3000	1500

Supplementary Tabel 2. Linking parameters for creating temporal V-shape trajectories are the search range and memory. To evaluate the quality of the V-shape trajectories, minimum and maximum temporal lengths are defined. Twice the size of the DRA averaging window is used as the maximum temporal lengths in the last column.

decoder instead of the standard up-sampling layer. This reduces edge artifacts during frame reconstruction. The outcome of the pre-trained network is a predicted frame ($\text{Frame}_{\text{predict}}$) and is subtracted from the frame under analysis to extract anomalous features (i.e., PSFs). These features are reshaped in order to be fed to iForest for further classification. The output of iForest is reshaped according to Section 5 in order to produce a 2D binary mask (Supplementary Fig. 9d). This mask is then convolved with a Gaussian with a half-width at half maximum of 2.5 pixels, corresponding to our experimental PSF size. Next, we check whether a connected area is created for a set of pixels engulfed within a PSF area. As a criterion for defining connectivity, we followed Otsu's thresholding algorithm[19]. Here, one constructs an intensity histogram for the pixels within a region of interest. This might result in several clusters, which are recognized by the thresholding algorithm. Next, we find the center of mass of each connected region and apply a binary mask with a radius of 5 pixels at the localized center of mass to represent where the expected protein could reside (Supplementary Fig. 9e). As a last step, the outcome is fed to DoG for localizing the PSFs in the DRA-treated video. Supplementary Fig. 9 illustrates the flow chart.

7 Temporal signature of the protein landing events

In an ideal scenario, landing particles generate a signal on the camera much like a step function. However, since we integrate over many frames to reach a detectable SNR, a differential rolling averaging (DRA) process yields a V-shaped curve as a function of time. The width of this V-profile is twice the DRA window averaging size. In figure 10, we show some examples of the V-shaped trajectories for the landing events of 9-21 kDa. Since there is a finite fluctuating background, only the values of the V-shape that are above the noise level (SNR>1) can be localized. The blue vertical dashed lines in figure 10 show the temporal time stamps, where the PSF was localized by our algorithm. We then temporally extended the V-shape of the localized event to determine the baseline. The red dashed lines in figure 10 correspond to twice the DRA window size and are centered at the peak values of the V-shapes. Moreover, in order for the reader to gain a better understanding of the V-shape process, we display the localized PSF of two landing events from the 9 kDa protein sample at different time stamps of t_{0-4} in figures 10m, n.

To compare a detected particle's landing event with normal temporal trajectories in the same videos, we removed the localized PSFs from the field of view and then randomly registered 770 pixels within 20 different iSCAT measurements. Exemplary contrast traces for such pixels are shown in figure 11a-c. One finds that the profiles do not exhibit a V-shaped landing signature (compare with landing events in figure 10). In other words, our algorithm is not able to fit two lines for extracting the contrast. Nevertheless, we have looked at the extremum of the trace and registered it as a contrast. For comparison, we have overlaid the histograms of true detection events and of the randomly selected events for the 9 kDa sample in figure 11d. We have repeated the same procedures for the protein samples of 18 kDa, and 21 kDa; the results are depicted in figures 11e-f respectively. Notably, the histograms of random pixels are much wider than the ones reported for the real events. This clearly shows that random selection does not have the spatial, temporal or the nominal features of a real landing event.

8 Performance of DNN vs user-defined feature matrix

In order to compare the performances of AD approaches based on DNN and a user-defined feature matrix, we syntactically altered the SNR of iSCAT PSFs. For this purpose, a binding iSCAT video of large proteins (ClpB hexamer, MW. 580 kDa) is selected and a DRA operation with batch sizes of 100, 500 and 2000 is applied. Then the PSFs are localized using AD based on DNN and user-defined feature matrix. It can be seen that at low SNRs (Supplementary Fig. 12c), DNN (Supplementary Fig. 12i) succeeds to identify the PSF while the user-defined feature matrix (Supplementary Fig. 12l) starts producing false positives.

Supplementary table 3 shows the statistics of the two algorithms regarding the detection yield of samples with 9, 18, and 21 kDa. The columns “Protein” and “Blank” present the normalized number of detections per million video frames for

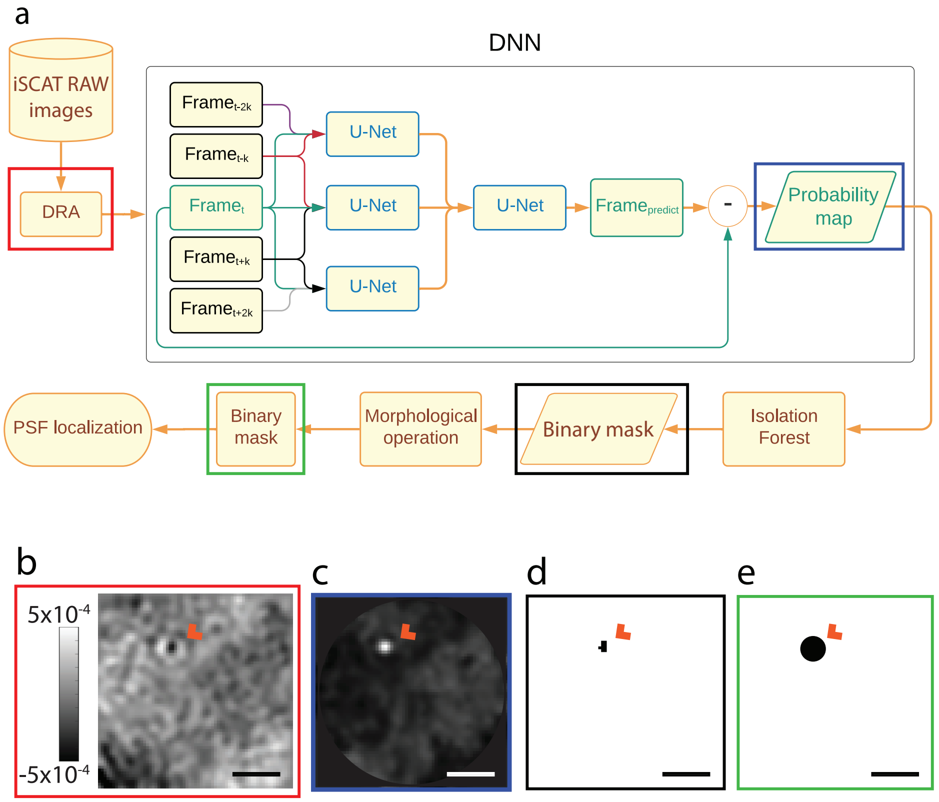

Supplementary Fig. 9. Anomaly Detection based on DNN. **a**, DRA operation is applied on iSCAT raw data. In order to localize the PSFs within each frame of the DRA, a series of identical U-nets are used. Next, Frame$_t$ is compared to the neighboring backward and forward frames for anomalies. Sets of Frames$_{t,\ t\text{-}k,\ t\text{-}2k}$, Frames$_{t,\ t\text{-}k,\ t\text{+}k}$, and Frames$_{t,\ t\text{+}k,\ t\text{+}2k}$ are each fed to an individual U-net. The result of each U-net is then fed to another U-net. The outcome is a predicted frame (Frame$_{predict}$) corresponding to time stamp t. It is then subtracted from Frame$_t$ to extract anomalous features (PSFs of proteins). These features are fed to iForest for further classification and PSF localization. **b**, DRA-treated iSCAT data with the red arrow pointing at a BSA protein. **c**, Sample frame, illustrating the output of DNN for the same PSF in (**c**). **d**, Result of iForest (binary mask) based on the probability map in (**c**). **e**, Hot region which is constructed based on the binary mask in (**d**). Scale bars are set to 1.5 μm. All figures are color coded accordingly.

experimental data after injecting a sample containing proteins or only a blank buffer, respectively. The column "Protein/Blank" depicts the ratio of the two resulting quantities. For example, DNN finds 9.7 times more proteins than false positive signals for a 9 kDa sample, whereas user-defined anomaly detection achieves 2.4.

Sample (kDa)	DNN			User Defined		
	Protein	Blank	Protein/Blank	Protein	Blank	Protein/Blank
9	81.5	8.5	9.7	20.8	8.5	2.4
18	55.4	3.8	14.6	33.9	13.5	2.6
21	167	8.1	20.5	207	37.8	5.4

Supplementary Tabel 3. DNN versus user-defined anomaly detection. Normalized number of detected proteins per million acquired frames. A total of 2.49, 4.75, and 1.93 million frames are acquired after injection of protein samples of 9, 18, and 21 kDa. For the control measurements, a total of 0.35, 0.52, and 0.37 million frames were acquired, respectively.

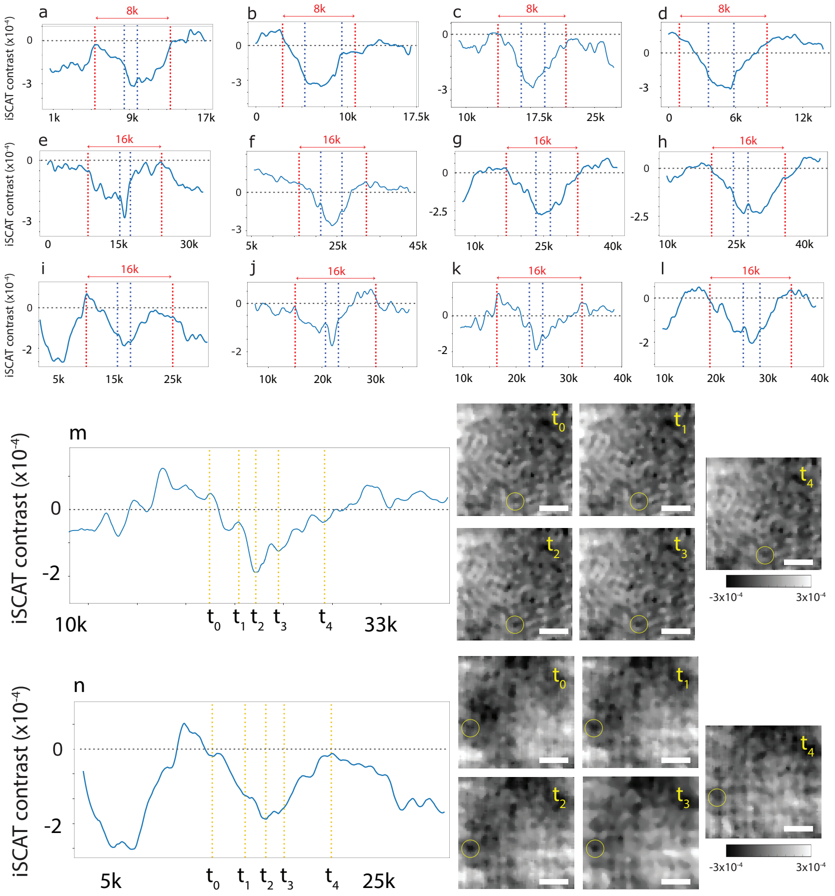

Supplementary Fig. 10. V-profile of the landing events of 21 kDa (a-d), 18 kDa (e-h), and 9 kDa (i-l) protein samples as a function of the number of frames. The blue vertical dashed lines show the temporal boundaries of the location where the PSF is detected. The trace is temporally extended beyond the boundaries in order to fit the V-Shape. m, n) Sample frames depicting the localized PSF shapes at time stamps of t_{0-4} for landing events of 9 kDa protein sample. The respective PSFs are engulfed by the yellow circles. The baseline at y=0 is shown using black dashed lines. The red dashed lines are twice the DRA batch size and are centered at the peak of the V-shapes. The x-axes are frame numbers. Scale bars equate to 1.5 μm.

9 Synthetic data

A set of synthetic data in a 70x70 pixel field of view and 40000 frames in length are generated. To produce a realistic speckle-like background, we used an experimentally recorded iSCAT background from a blank cover glass. We then generated 2D Gaussian spots with the same nominal contrast as expected for the 9-66 kDa protein to mimic the PSF of a protein. Here, we used experimentally recorded frames of the cover glass in the absence of proteins as background. We then multiplied this background with the expected contrast of a PSF at the desired location. We note that since the background comes from experimental data, it already encompasses several noise sources including shot noise (Supplementary Fig. 13a). We fabricated 40 videos, each containing four particles and random starting frame numbers (i.e., start of the landing event). All particles remain stationary until the end of the video, and there is no desorption. The location of a synthetically added particle is depicted in Supplementary Fig. 13a. Supplementary Fig. 13b shows a typical V-shaped landing event.

10 Photobleaching and interlaced iSCAT-TIRF measurements

We could not perform simultaneous iSCAT and TIRF measurements because we observed strong photobleaching. To elaborate on the photophysics, in Supplementary Fig. 14 we present the bleaching dynamics for labeled protein samples of ultra-pure bovine serum albumin (a, e), recombinant protein G (b, f), and recombinant human IL-8 protein (c, g). The on-time of each fluorophore is inserted as a label in units of *ms* right next to its image. To demonstrate the effect of the iSCAT laser (1mW/μm^2) on the bleaching rate, we compare the fluorescence of the molecules in two scenarios, where the blue laser is switched off (Supplementary Fig. 14a-c) and on (Supplementary Fig. 14e-g). Supplementary Fig. 14d, and 14h depict the histograms of the

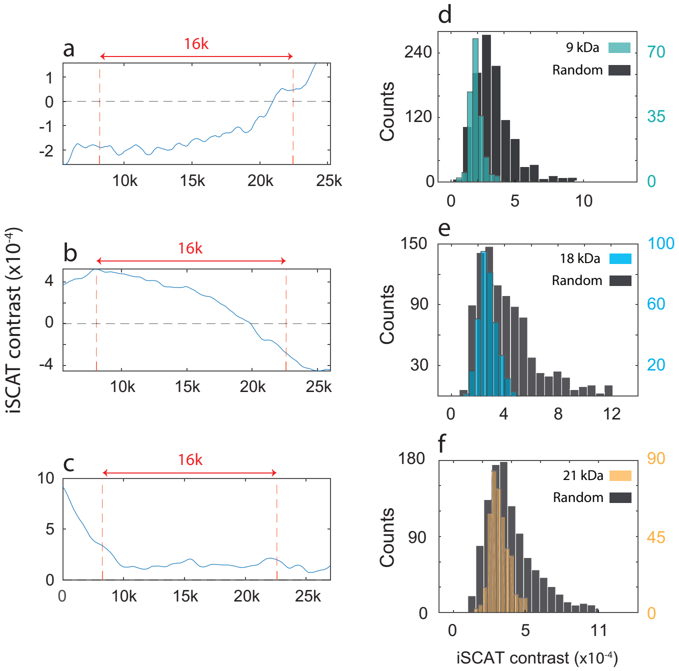

Supplementary Fig. 11. Random pixels further than a radius of 5 from the center of the localized 9 kDa protein PSFs are chosen and the temporal trace of the contrast is depicted in (a-c). d-f) Histogram of 770, 660, and 616 registered random pixels and comparison to the real binding events for the protein samples of 9, 18, and 21 kDa.

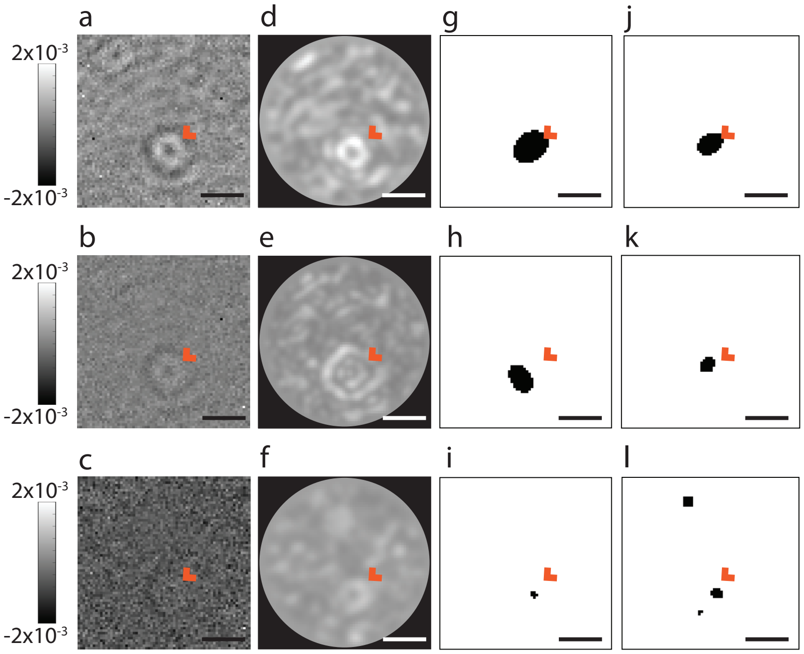

Supplementary Fig. 12. An example for the comparison of Anomaly localization based on DNN and user-defined feature matrix. iSCAT video of a large protein (ClpB hexamer) binding is selected and DRA operations with batch sizes of 2000 (**a**), 500 (**b**), and 100 (**c**) are applied. **d-f**, DNN probability map corresponding to the localized PSFs. **g-i**, binary mask generated based on DNN. **j-l**, Binary mask based on a user-defined feature matrix. Scale bars are 1.5 μm.

on-times. It is clear that the lifetime of the fluorophores is reduced by about three times in this case.

To further investigate the effect of the blue laser on fluorescent labels, we conducted a series of measurements, where BSA molecules labeled with Atto 647 were spin coated on a clean coverglass and immobilized. Here, we first illuminated the samples

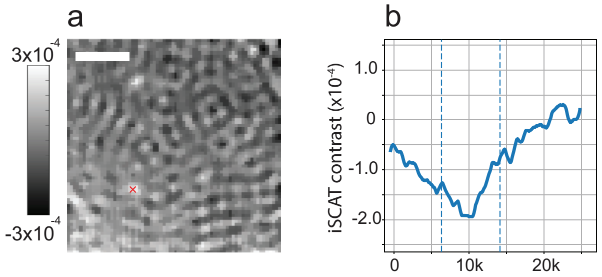

c

9 kDa	True	False	GT	Accuracy (%)	Yield (%)	False Positive
User defined	78	10	160	88%	49%	12%
DNN	91	8	160	92%	57%	8%

d

21 kDa	True	False	GT	Accuracy (%)	Yield (%)	False Positive
User defined	90	8	160	92%	56%	8%
DNN	109	6	160	95%	68%	5%

e

66 kDa	True	False	GT	Accuracy (%)	Yield (%)	False Positive
User defined	118	0	160	100%	74%	0%
DNN	144	0	160	100%	90%	0%

Supplementary Fig. 13. Synthetic landing of protein samples. a) Sample frame of the corresponding synthetic landing, with a DRA batch size of 7000 frames. Scale bar is 1.5 μm. b) V-shape profile of a synthetic 9 kDa object. c-e) Performance of the localization algorithm on the 9, 18, and 66 kDa synthetic data sets. GT is the total number of generated particles. Accuracy is defined as the number of true positives divided by the total number of detections. Yield is defined as the number of true positives over the total number of generated particles.

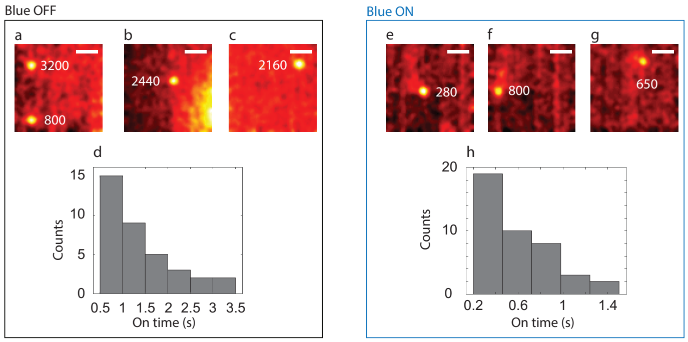

Supplementary Fig. 14. Bleaching rate of the fluorophores. a-c) Snap shot of different fluorophores under TIRF illumination. e-g) Sample frames, depicting the fluorophores while the iSCAT laser (λ= 445 nm) was switched on. d, h) Histogram of the fluorescence decay rates while the blue laser is switched off and on respectively. White labels show the on time of each fluorophore in ms. Scale bars are 1.5 μm.

using the 631 nm laser and recorded the emission for a fraction of a second to obtain the location of the molecules. We then illuminated the sample for a period of 6 s with the 445 nm blue laser at the usual power for conducting iSCAT measurements. Next, we switched on the red laser again to see how many molecules have survived. We find that the majority of the molecules are not bleached. Supplementary Fig. 15a displays the results.

In another series of measurements on the same aforementioned sample, we quantified the effect of the blue laser on already excited molecules. We quantified the decay rates where only the red laser was on (Supplementary Fig. 15b) and when the blue laser illuminated the molecules (Supplementary Fig. 15c). We observe that when the molecules are already excited via the red laser, the presence of the blue laser light drastically reduces the life time to below 1 s. However, when only the blue laser is on, the molecules are not excited and therefore not bleached (Blue On row in Supplementary Fig. 15a). These results are in alignment with the findings of Supplementary Figs. 14d, and h.

To get around the photobleaching problem, we performed interlaced iSCAT and TIRF measurements in the following

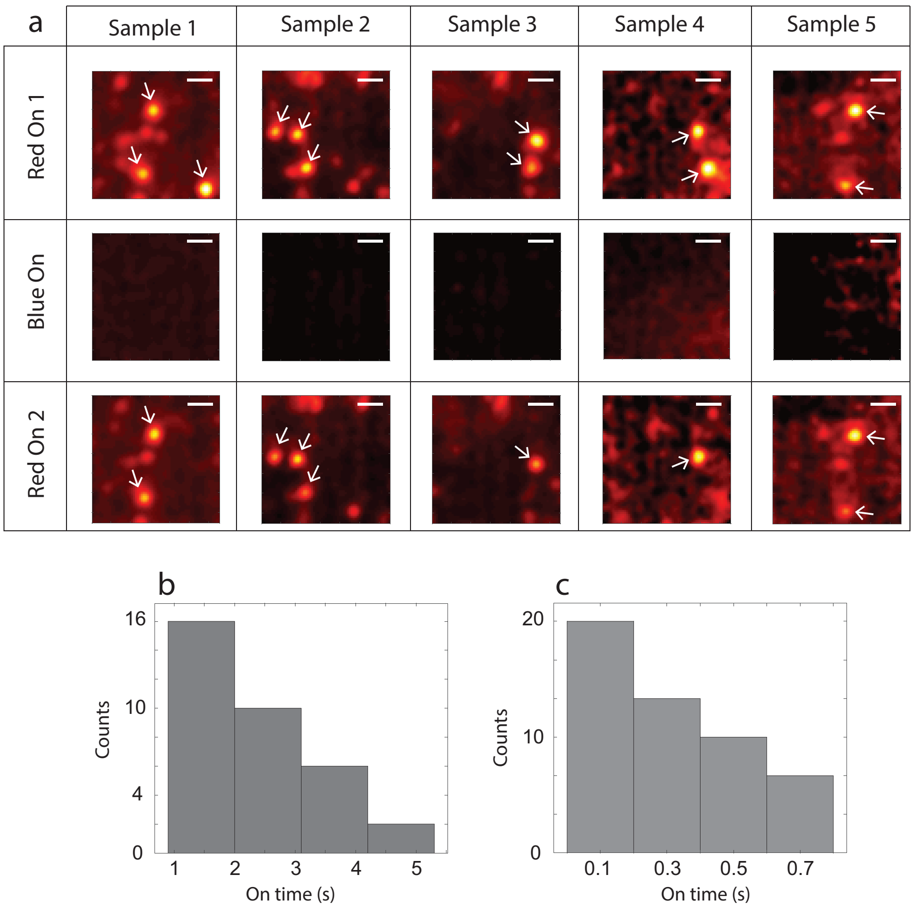

Supplementary Fig. 15. a) Fluorescence emission of labelled BSA molecules. Red On 1 reports on measurements when the sample is illuminated with the red laser for a short period of time. Blue On shows measurements when the sample is only illuminated with the 445 nm laser light. Red On 2 shows the case, where the sample is excited right after the blue laser is switched off. b) Bleaching rate when the molecules are excited using the red laser (631 nm). c) Bleaching rate when the blue laser is also switched on. White arrows point to the labeled proteins with scale bars equating to 1.5 μm.

manner: A certain amount of protein is injected, the laser beam at $\lambda = 445$ nm and the CMOS camera in the iSCAT channel are triggered (ie. T1 in Supplementary Fig. 16a), and a series of consecutive frames are acquired for a period of 6-10 s, depending on the frame rate of the camera. Next, the blue laser is switched off, and the red laser at $\lambda = 631$ nm as well as the TIRF camera are immediately triggered (ie. T2 in Supplementary Fig. 16a) to acquire 5 s of TIRF measurements. This procedure is repeated for several cycles of protein injection and interlaced data acquisition in order to reach meaningful statistics of the localized iSCAT events. Supplementary Fig. 16a shows the scheme of the interlaced measurements.

In Supplementary Figs. 16b-d, we present an example of data from a protein sample of 21 kDa. The iSCAT and TIRF localized events during one of the interlaced cycles are depicted. The histograms in figures 5 c-e of the main text report on the events that are corroborated by both iSCAT and TIRF (i.e., Supplementary Fig. 16e). The coincidence of the main peaks of group 1 and group 2 provides a strong evidence that the fidelity of the analysis is not compromised although the efficiency of the iSCAT and TIRF coincidence suffers from the fact that the measurements are not simultaneous.

To provide more insight into the problem of coincidence between iSCAT and TIRF images, we truncated our iSCAT data and looked at only the last 1 second of each measurement. We then registered the localized events and compared them against the proceeding TIRF measurements. This reduces the statistics, but Supplementary Fig. 17d-f reveals a large increase in the coincidence between the iSCAT and TIRF data. Supplementary table 4 summarizes the number of registered events for each histogram. The main modes of the histograms remain well aligned.

In Supplementary Fig. 18, we present evidence for another phenomenon that can affect the quality of coincidences between the iSCAT and TIRF channels: Proteins might move around after initial contact with the substrate and in some cases also desorb after a few seconds. Examples for the mobility of ultra-pure bovine serum albumin (a-b), recombinant protein G (c-d), and recombinant E. Coli Skp protein (e-f) are presented.

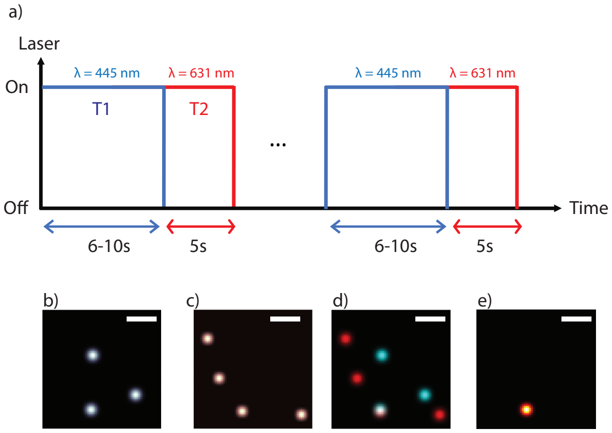

Supplementary Fig. 16. a) Interlaced scheme of iSCAT and TIRF measurements. T1 triggers the blue laser at λ= 445 nm for a period of 6-10 s. After switching off the blue laser, T2 switches on the red laser (λ= 631 nm) for conducting the corresponding TIRF measurement with a duty cycle of 5 s. b) Localized iSCAT events where a 21 kDa protein sample is injected (Recombinant protein G). c) Localized TIRF incidences of the same protein sample. d) overlay of the iSCAT and TIRF events. e) pixel wise cross correlation of the TIRF and iSCAT channel. Scale bars are 1.5 μm.

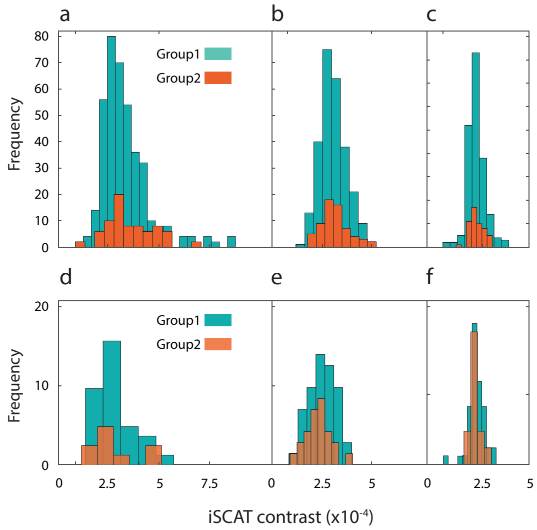

Supplementary Fig. 17. Correlation of iSCAT and TIRF events. Protein landing events of a) Recombinant protein G (21 KDa), b) Recombinant E. Coli Skp protein (18 KDa), and c) Recombinant human IL-8 protein (9 kDa). d-e, landing events which took place during the last 1 second of iSCAT measurements for the 21 kDa (d), 18 kDa (e), and 9 kDa (f) protein samples. Group1 corresponds to events which took place solely during iSCAT and Group2 represents the ones which were also localized during the proceeding TIRF measurement.

11 False positive detection

Depending on the molecular mass of the protein, we typically register between 0.2 and 2 proteins per 1 s while we observe a rate of 1-5 proteins per second in the TIRF channel. This shows that we do not over-count. Nevertheless, we performed simulations to investigate the probability of including background noise in our detection counts. We generated a series of synthetic data to simulate the binding events of 9, 21, and 66 kDa protein samples to the surface. We find that the user-defined algorithm reaches an accuracy of 88-100% with a yield of 49-74%. The DNN approach does even better with an accuracy level of 92-100% and yield of 57-90%. The details of the synthetic data generation can be found in supplementary section 9. This provides further evidence that false positive rates are always less than about 10%. The observed yield or false negative events can somewhat skew the overall information about the number of detected particles or concentrations. We remark, however, that the quality of mass measurements, which is of central interest in the new field of mass photometry, is not compromised.

Sample (kDa)	DNN (full)	TIRFxDNN (full)	Coincidence Yield (full)	DNN (1s)	TIRFxDNN (1s)	Coincidence Yield (1s)
21	322 (Group1, Fig4a)	84 (Group2, Fig4a)	26%	45 (Group1, Fig4d)	16 (Group2, Fig4d)	35%
18	263 (Group1, Fig4b)	81 (Group2, Fig4b)	31%	42 (Group1, Fig4e)	23 (Group2, Fig4e)	55%
9	201 (Group1, Fig4c)	55 (Group2, Fig4c)	27%	48 (Group1, Fig4f)	26 (Group2, Fig4f)	54%

Supplementary Tabel 4. Number of registered particles for each histogram in Supplementary Fig.17 is listed. DNN (full) shows the number for all the registered particles during iSCAT. TIRFxDNN (full) shows the number of particles that were both present in iSCAT and TIRF. Coincidence yield (full) shows the percentage of particle that correlated divided through all the iSCAT events. DNN (1s) shows the number of registered particles during the last 1 second of all iSCAT measurements. TIRFxDNN (1s) corresponds to the correlation between the last 1s of iSCAT with the proceeding TIRF measurements. Yield (1s) depicts the corresponding ratio of iSCAT to TIRF events.

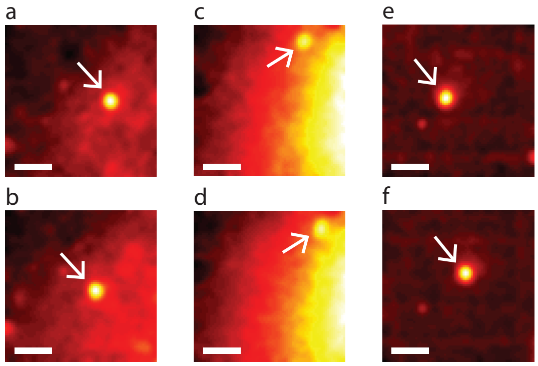

Supplementary Fig. 18. Protein mobility. Sample frames depicting protein movement after binding to the coverglass during a period of a-b) 200 ms, c-d) 240 ms, e-f) 280 ms. The white arrows point to the fluorescent molecules. Scale bars are 1.5 μm.

12 Tuning iForest

We have examined the effect of contamination score on the shape of the histograms of the lowest SNR protein (9 kDa) sample. Here, we evaluated the performance of iForest by changing the contamination size and examined how it affects the output of DNN. In our usual analysis, we use contamination scores in the range 0.002-0.003 based on synthetically generated data and by examining the data obtained from large proteins (e.g., 66 kDa). For contamination scores below 0.001, we found that iForest was not able to detect many particles, whereas for scores above 0.01, the number of detected pixels increases dramatically, preventing one from isolating individual landing events. In Supplementary Figure 19, we present the results for three different contamination scores in the range 0.001-0.006. We find that the histogram peak position remains unchanged. However, the choice of the parameters can affect the yield, following a general trend that a tighter histogram comes at the cost of a lower yield. In other words, one adjusts the upper bound of the contamination score to prevent false detections and sets the lower bound to allow for a sufficient success rate or yield. When studying unknown samples, one can run measurements on a series of concentrations to check that the selected contamination score results in the same trend in the observed detection events as in the dilution process.

13 Training time and computational power

In order to train the network and converge to the optimal set of weights, we started from random values and then carried out the training over 10 different sets of videos with a size of 70x70 pixels, 75 K frames long and over 25 epochs. We utilized a workstation equipped with RTX6000 (GPU) and Threadripper PRO 3995WX (CPU) and the training task took about 1050 minutes. After training, the analysis for each video (with the aforementioned size) took about 21 minutes.

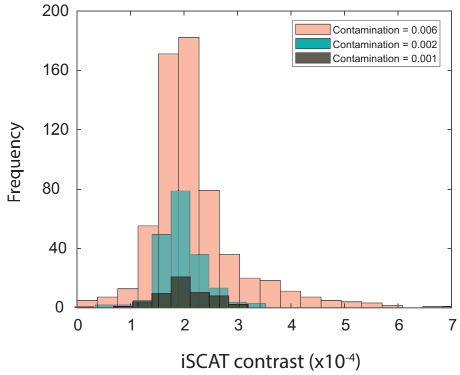

Supplementary Fig. 19. Hyper parameter tuning. The contamination score of iForest is modulated between 0.001-0.006 and the histograms are depicted.

References

1. Dastjerdi, H. M. *et al.* Optimized analysis for sensitive detection and analysis of single proteins via interferometric scattering microscopy. *J. Phys. D.* **55**, 054002 (2021).
2. McLachlan, G. J. & Peel, D. *Finite mixture models* (John Wiley & Sons, 2004).
3. Murphy, K. P. *Machine learning: a probabilistic perspective* (MIT press, 2012).
4. Hastie, T., Tibshirani, R. & Friedman, J. *Model Assessment and Selection*, chap. 7, 235 (Springer, 2016).
5. Bishop, C. M. & Nasrabadi, N. M. *Pattern recognition and machine learning*, vol. 4 (Springer, 2006).
6. Dunning, T. & Friedman, E. *Practical machine learning: a new look at anomaly detection* (" O'Reilly Media, Inc.", 2014).
7. Chandola, V., Banerjee, A. & Kumar, V. Anomaly detection: A survey. **41**, 58 (2009).
8. Liu, F. T., Ting, K. M. & Zhou, Z.-H. Isolation forest. In *2008 Eighth IEEE International Conference on Data Mining*, 413–422 (2008).
9. Warr, K. *Strengthening deep neural networks: Making AI less susceptible to adversarial trickery* (O'Reilly Media, 2019).
10. Ronneberger, O., Fischer, P. & Brox, T. U-net: Convolutional networks for biomedical image segmentation. In Navab, N., Hornegger, J., Wells, W. M. & Frangi, A. F. (eds.) *Medical Image Computing and Computer-Assisted Intervention – MICCAI 2015*, 234–241 (Springer International Publishing, Cham, 2015).
11. Ioffe, S. & Szegedy, C. Batch normalization: Accelerating deep network training by reducing internal covariate shift. In *International conference on machine learning*, 448–456 (PMLR, 2015).
12. Shi, W. *et al.* Real-time single image and video super-resolution using an efficient sub-pixel convolutional neural network. In *Proceedings of the IEEE Conference on Computer Vision and Pattern Recognition (CVPR)* (2016).
13. Mao, X., Shen, C. & Yang, Y.-B. Image restoration using very deep convolutional encoder-decoder networks with symmetric skip connections. In *Advances in Neural Information Processing Systems* (Curran Associates, Inc., 2016).
14. Tassano, M., Delon, J. & Veit, T. Fastdvdnet: Towards real-time deep video denoising without flow estimation. In *Proceedings of the IEEE/CVF Conference on Computer Vision and Pattern Recognition (CVPR)* (2020).
15. Voss, N., Yoshioka, C., Radermacher, M., Potter, C. & Carragher, B. DoG Picker and TiltPicker: software tools to facilitate particle selection in single particle electron microscopy. *J. Struct. Biol.* **166**, 205–213 (2009).
16. Ting, K., Liu, F. & Zhou, Z. Isolation forest. In *IEEE International Conference on Data Mining, ICDM 2008*, 413–422 (IEEE, 2008).
17. Tassano, M., Delon, J. & Veit, T. Fastdvdnet: Towards real-time deep video denoising without flow estimation. In *Proceedings of CVPR*, 1354–1363 (2020).
18. Shi, W. *et al.* Real-time single image and video super-resolution using an efficient sub-pixel convolutional neural network. In *Proc. IEEE Int. Conf. Comput. Vis.*, 1874–1883 (2016).
19. Bangare, S. L., Dubal, A., Bangare, P. S. & Patil, S. Reviewing otsu's method for image thresholding. *Int. J. Appl. Eng* 21777–21783 (2015).
