# Supplementary material for: Self-supervised machine learning pushes the sensitivity limit in label-free detection of single proteins below 10 kDa
Source: Nat Methods. 2023 Feb 27;20(3):442–7. doi: 10.1038/s41592-023-01778-2 (PMC9998267; doi:10.1038/s41592-023-01778-2)
Supplement: Supplementary file 2 — Reporting Summary [file 41592_2023_1778_MOESM2_ESM.pdf]

nature portfolio

Corresponding author(s): Vahid Sandoghdar

Last updated by author(s): 17.12.2022

# Reporting Summary

Nature Portfolio wishes to improve the reproducibility of the work that we publish. This form provides structure for consistency and transparency in reporting. For further information on Nature Portfolio policies, see our Editorial Policies and the Editorial Policy Checklist.

## Statistics

For all statistical analyses, confirm that the following items are present in the figure legend, table legend, main text, or Methods section.

| n/a | Confirmed | |
|---|---|---|
| ☐ | ☒ | The exact sample size (*n*) for each experimental group/condition, given as a discrete number and unit of measurement |
| ☐ | ☒ | A statement on whether measurements were taken from distinct samples or whether the same sample was measured repeatedly |
| ☒ | ☐ | The statistical test(s) used AND whether they are one- or two-sided<br>*Only common tests should be described solely by name; describe more complex techniques in the Methods section.* |
| ☒ | ☐ | A description of all covariates tested |
| ☒ | ☐ | A description of any assumptions or corrections, such as tests of normality and adjustment for multiple comparisons |
| ☐ | ☒ | A full description of the statistical parameters including central tendency (e.g. means) or other basic estimates (e.g. regression coefficient) AND variation (e.g. standard deviation) or associated estimates of uncertainty (e.g. confidence intervals) |
| ☒ | ☐ | For null hypothesis testing, the test statistic (e.g. *F*, *t*, *r*) with confidence intervals, effect sizes, degrees of freedom and *P* value noted<br>*Give P values as exact values whenever suitable.* |
| ☒ | ☐ | For Bayesian analysis, information on the choice of priors and Markov chain Monte Carlo settings |
| ☒ | ☐ | For hierarchical and complex designs, identification of the appropriate level for tests and full reporting of outcomes |
| ☒ | ☐ | Estimates of effect sizes (e.g. Cohen's *d*, Pearson's *r*), indicating how they were calculated |

*Our web collection on statistics for biologists contains articles on many of the points above.*

## Software and code

Policy information about availability of computer code

| | |
|---|---|
| Data collection | pyLabLib Cam-control was used to collect the iSCAT data, and HoKaWo (Hamamatsu photonics) was used to record TIRF measurements. |
| Data analysis | The software package used in this study is freely available at https://piscat.readthedocs.io/. |

For manuscripts utilizing custom algorithms or software that are central to the research but not yet described in published literature, software must be made available to editors and reviewers. We strongly encourage code deposition in a community repository (e.g. GitHub). See the Nature Portfolio guidelines for submitting code & software for further information.

## Data

Policy information about availability of data

All manuscripts must include a data availability statement. This statement should provide the following information, where applicable:

- Accession codes, unique identifiers, or web links for publicly available datasets
- A description of any restrictions on data availability
- For clinical datasets or third party data, please ensure that the statement adheres to our policy

The data is available upon reasonable request. Due to the large size of our data (~10 TB), we cannot release them publicly, but they are available upon request. The code which is used for data analysis is made public at https://github.com/SandoghdarLab. Also, the results of the analysis, which yielded figures 5c-e and 6, are available as well.

## Human research participants

Policy information about studies involving human research participants and Sex and Gender in Research.

| | |
|---|---|
| Reporting on sex and gender | NA |
| Population characteristics | NA |
| Recruitment | NA |
| Ethics oversight | NA |

Note that full information on the approval of the study protocol must also be provided in the manuscript.

# Field-specific reporting

Please select the one below that is the best fit for your research. If you are not sure, read the appropriate sections before making your selection.

☒ Life sciences ☐ Behavioural & social sciences ☐ Ecological, evolutionary & environmental sciences

For a reference copy of the document with all sections, see nature.com/documents/nr-reporting-summary-flat.pdf

# Life sciences study design

All studies must disclose on these points even when the disclosure is negative.

| | |
|---|---|
| Sample size | A total of 2.49, 4.75, and 1.93 million frames are acquired and processed to form the histograms of the 9, 18, and 21 kDa protein samples, respectively. At least 200 single events are detected for each protein type and used to form an iSCAT contrast histogram. |
| Data exclusions | No data were excluded from the analysis. |
| Replication | Figs. 1b-c are snapshots of a single experiment. Fig. 2 shows a binding event for a single 66 kDa protein. Fig. 3 shows single events; each iSCAT binding event is deduced from averaging thousands of frames of an iSCAT measurement by a DRA treatment (as explained in the main text). Fig. 4 is a single iSCAT measurement. Figs. 5a-b are snapshots of a single experiment. Figs. 5c-e are a collection of 322, 263, and 201 data points, each corresponding to a single binding event collected over 1.93, 4.75, and 2.49 million frames, respectively. Fig. 6a is the result of a corresponding procedure for each protein type/size. |
| Randomization | The error bars in Fig. 6a are a result of bootstrapping that inherently contains randomization. |
| Blinding | The results of this study are based on collecting hundreds of single-protein events. We have performed TIRF measurements and synthetic data to benchmark the analysis pipeline. The aforementioned synthetic data were generated in a blind fashion. In other words, the timestamp of the protein arrivals at the field of view was unknown. Blinding in the experimental measurements would also be possible, but was not deemed necessary in our study. |

# Reporting for specific materials, systems and methods

We require information from authors about some types of materials, experimental systems and methods used in many studies. Here, indicate whether each material, system or method listed is relevant to your study. If you are not sure if a list item applies to your research, read the appropriate section before selecting a response.

### Materials & experimental systems

| n/a | Involved in the study | |
|---|---|---|
| ☒ | ☐ | Antibodies |
| ☒ | ☐ | Eukaryotic cell lines |
| ☒ | ☐ | Palaeontology and archaeology |
| ☒ | ☐ | Animals and other organisms |
| ☒ | ☐ | Clinical data |
| ☒ | ☐ | Dual use research of concern |

### Methods

| n/a | Involved in the study | |
|---|---|---|
| ☒ | ☐ | ChIP-seq |
| ☒ | ☐ | Flow cytometry |
| ☒ | ☐ | MRI-based neuroimaging |
